# ‘I think it affects every aspect of my life, really’: Cancer survivors’ experience of living with chronic pain after curative cancer treatment in England, UK

**DOI:** 10.1371/journal.pone.0290967

**Published:** 2023-09-01

**Authors:** Julie Armoogum, Claire Foster, Alison Llewellyn, Diana Harcourt, Candida McCabe

**Affiliations:** 1 College of Health, Science and Society, University of the West of England, Bristol, United Kingdom; 2 Centre for Psychosocial Research in Cancer, School of Health Sciences, University of Southampton, Southampton, United Kingdom; 3 Dorothy House Hospice, Winsley, United Kingdom; University of Sharjah, UNITED ARAB EMIRATES

## Abstract

**Aim:**

To explore cancer survivors’ experiences of living with chronic pain after curative cancer treatment in England, UK.

**Methods:**

A qualitative study using telephone interviews with adult cancer survivors experiencing chronic pain after curative cancer treatment. Interview data was analysed using a reflexive thematic approach [1–3].

**Findings:**

Nineteen participants: 14 female, 5 male, mean age 62.4 years, 1.5–48 years since cancer diagnosis, eight tumour groups represented. Six participants (31.6%) developed chronic pain more than ten years after completing cancer treatment (range 0–25 years). Five themes were generated which highlighted the experience of chronic pain after cancer treatment for cancer survivors: 1) ‘Hear me… believe me…. Please’. Survivors felt that they had not been listened to when they tried to talk about their chronic pain after cancer treatment, nor at times, believed. 2) ‘Expectation versus reality’. Survivors had anticipated returning to pre cancer quality of life yet living in chronic pain prevented them from doing so. 3) ‘They don’t understand…. We don’t understand’. Cancer survivors did not feel informed or prepared for the risk or reality of chronic pain after cancer treatment and this compounded the difficulties of coping with and managing their pain. They felt health care professionals lacked knowledge and understanding of chronic pain after cancer. 4) ‘Negotiating the maze’. Cancer survivors encountered unclear and limited pathways for support, often bouncing from one support team to another. Identifying and accessing services was a challenge, and the responsibility of this was often left to the survivor. 5) ‘Validate my pain, validate me’. Palpable relief and benefit was felt when health care professionals diagnosed and acknowledged their chronic pain after cancer treatment.

**Conclusions:**

Cancer survivors can feel ill prepared for the risk of chronic pain after cancer treatment and can experience challenges accessing support from healthcare professionals and clinical services.

## Introduction

Survival rates for cancer are improving and more people are living for longer following their cancer treatment [4, 5]. Approximately half of all people diagnosed with cancer will survive for ten years or more [6]. In 2018, there were 43.8 million people living with cancer worldwide who were diagnosed in the last five years [7] and within the UK, it is predicted there will be four million cancer survivors by 2030 [5]. Whilst cancer survival rates are increasing, it is known that not everyone is living well after curative cancer treatment and people can experience many physical and psychological symptoms [8–10]. These symptoms can be long-term treatment effects (i.e. side-effects that begin during and extend beyond treatment completion) and also late effects, which occur months or years after treatment is finished [5]. Chronic post cancer treatment pain is a common long term or late effect of cancer treatment. Globally, prevalence rates of people experiencing pain after curative cancer treatment are reported as approximately 39% [11] but some studies have found as many as 72% experience pain after treatment has ended [12]. However, until recently, there has not been a method of formally recognising, coding or systematically recording chronic cancer related pain [13]. In 2019, the International Association for the Study of Pain (IASP) and the World Health Organisation (WHO) devised new classifications for the International Classification of Diseases-11 (ICD-11) for chronic cancer related pain. This recognised that the main sources for cancer related chronic pain are the cancer itself and also *the treatments used to combat it* [13]. Thus, the IASP introduced a specific classification for chronic post-cancer treatment pain defined as pain that lasts for 3 months or more and caused by cancer treatments, such as chemotherapy, radiotherapy or surgery [13]. For many cancers, surgery is the principal treatment and chronic or persistent pain may occur after any surgical procedure [14]. Although chronic post-surgical pain can follow any surgery it is most strongly associated with thoracotomy, limb amputation and breast surgery [14, 15]. Radiotherapy is a key aspect of cancer treatment. Damage is affected by the dose, volume of tissue affected and the susceptibility of the tissue to radiation. Nervous tissue is vulnerable to damage from ionising radiation but there are also secondary and longer term affects arising from fibrosis and vascular changes [14]. Chemotherapy and other systemic anti-cancer treatments such as hormone and biological therapies can also cause chronic pain. Most well recognised is chemotherapy induced peripheral neuropathy [CIPN], a chronic peripheral neuropathic pain caused by oral or intravenous chemotherapy given to treat primary disease or metastases [13] but chronic pain can also arise from graft versus host disease (GVHD) after allogeneic stem cell transplant in haematological malignancy and others [9]. Herein pain that lasts for 3 months or more and caused by cancer treatments, such as chemotherapy, radiotherapy or surgery, will be referred to as chronic pain after cancer treatment.

The EORTC (European Organisation for the Research and Treatment of Cancer) defines ‘cancer survivor’ as any person diagnosed with cancer, who has completed his or her primary treatment (with the exception of maintenance therapy), and who has no active disease [16]. There are reports of high unmet needs in cancer survivors living with pain: it was one of the most prevalent moderate to severe unmet needs in cancer survivors 24 months after colorectal cancer surgery (n = 510) [17] and the third most frequent physical unmet need (19%) among 625 breast cancer survivors [18]. Cancer survivors who live with pain are more likely to report feeling depressed, worried, nervous or anxious, being unable to work, and needing assistance with activities of daily living [19]. This highlights that pain is a significant problem for cancer survivors, yet it does not describe the nature of cancer survivors’ experiences of living with chronic pain after cancer treatment. Understanding experiences and needs of cancer survivors is key to improving how well people live after cancer [10].

There is a paucity of qualitative research exploring the experience of living with chronic pain after cancer treatment. Living with chronic pain in the non-malignant population is well described [20], however, there appears to be a bespoke element to the experience when the pain arises from cancer treatment [18]. From the limited evidence available exploring chronic pain after cancer treatment, cancer survivors consider their experience of chronic pain and cancer to be intrinsically interwoven [21]. Pain was viewed by many cancer survivors as an indicator of their current cancer status, representing their transitional state between health and illness and serving as a reminder of the threat that they had experienced to their mortality [18]. Published studies do not include the experiences of men, who are underrepresented in qualitative research generally [21, 22] Furthermore, the majority of studies examining pain after cancer focus on those in the early stages of survivorship from breast cancer [9–13] and yet chronic pain can occur as a late effect of treatment from many other forms of cancer [11, 23, 24].

Cancer survivors can feel abandoned when treatment finishes and struggle to understand what to expect from health care systems after cancer [25–27]. Challenges include difficulties regarding access, professional responsiveness, co-ordination, communication, involvement in care and workforce shortages [27–29] and these are exacerbated when the problems occur as late effects, often years after treatment has ended [24]. Cancer survivors may also lack confidence or feel vulnerable asking for help; or may not expect chronic pain to occur after treatment [30]. Thus, cancer survivors can face difficulty in obtaining the help they need for chronic pain. This is a worldwide issue. In Canada, Fitch et al. [2021] examined the experience of cancer survivors transitioning from cancer services to survivorship care. They reported 34% (n = 4058) experienced chronic pain and over a third of this population had trouble in obtaining help for their pain.

The principal research aim of this research was explore cancer survivors’ experiences of living with chronic pain after curative cancer treatment in England, UK.

## Methods

### Research design and theoretical framework

This qualitative study adopted an experiential theoretical framework. Therefore it concentrated on participants’ standpoints and how they saw the world and aimed to capture and explore participants’ own perspectives and understanding [31]. The research took a critical realist ontological perspective within the broad epistemological framework of contextualism. Thus the research captured participants’ experiences as lived realities that were produced, and existed, within broader contexts [3, 32]. National Health Service (NHS) Research Ethics and Health Regulatory Authority (HRA) approvals (19/NW/0405) and University of the West of England (UWE) Research Ethics approval (HAS.19.10.043) were obtained. A UWE Research Governance Record and Data Management Plan were maintained throughout the study.

### Public involvement

Two public contributors, who are cancer survivors living with chronic pain after cancer treatment, were recruited to be involved with the development, design and conduct of the study. Their involvement followed the UK Standards for public involvement in research [33, 34]. Feedback from public contributors resulted in modifications to the methods of the study including informing General Practitioners (GP) about participant involvement, wording of the recruitment poster and interview schedule, and including patient groups within the plans for dissemination. An evaluation of the impact of the public contributors followed the ‘cube’ framework for evaluation [35]. The CUBE framework acknowledges that public and patient involvement is dependent on context and involves interactions between different forms of knowledge (public, professional) within a ‘knowledge space’. It proports there are four dimensions of knowledge space (voices, involvement, concerns, change), each on a continuum [35]. The evaluation demonstrated that all members of the research team felt the public contributors had a strong voice within the project, they were involved in many ways and the organisation was willing to change. Full details of the evaluation will be published shortly.

### Sample framework

The sample were purposively recruited from an NHS service, two English support organisations and a research group, specifically: 1) Complex Cancer Late Effects Rehabilitation Service (CCLERS) at the Royal National Hospital for Rheumatic Diseases (RNHRD), Bath, (now known as the Pain-related Cancer Late Effects Functional Rehabilitation Service, Royal United Hospitals NHS Foundation Trust, Bath, UK)—an NHS cancer late effects service https://www.crpsandcancerlateeffects-bath.org.uk/ 2) Penny Brohn UK, a national cancer charity running support services for anyone affected by cancer https://www.pennybrohn.org.uk/**;** 3) Participants from a national cohort study, HORIZONS, exploring recovery of health and wellbeing following cancer treatment and run by the Centre for Psychosocial Research in Cancer (CentRIC^+^) at the University of Southampton http://www.horizons-hub.org.uk/index.html and 4) Radiotherapy Action Group Exposure [R.A.G.E], a support and campaigning group for those suffering injury from radiotherapy given as treatment for breast cancer https://www.rageuk.org/.

#### Inclusion criteria

- Adults over 16 years old when diagnosed and treated for cancer in England
- Self-reported cancer survivors as per EORTC definition (any person diagnosed with cancer, who has completed his or her primary treatment with the exception of maintenance therapy), and who has no active disease] [16]
- Self-reported chronic pain after cancer treatment
- People able to verbally communicate in the English language

#### Exclusion criteria

- Survivors of childhood cancer
- Previously seen by a chronic pain clinical team for non-malignant chronic pain

Recruitment was stratified by gender, cancer type and stage of cancer care pathway [36]. The main phases of the cancer care pathway include diagnosis and treatment, rehabilitation, monitoring, progressive illness and end of life [36]. Participants were recruited at the rehabilitation and monitoring stage of the cancer care pathway, thus if they were within a year, 5–10 years or beyond 10 years of completing curative cancer treatment [36]. When recruitment sites were identifying participants, men and those with non breast cancer, who met the inclusion criteria, were approached first. The four recruiting sites identified and approached potential participants. How participants were identified was specific to site depending on their referral procedures, standard measurements for pain and communication practices. Referral criteria to CCLERS includes severe or persistent pain as a consequence of cancer treatment. To identify participants for this study, the CCLERS team reviewed referral lists for the past two years to identify potential participants who met the criteria. Penny Brohn UK reviewed registration forms of clients and identified clients who had highlighted pain as a concern in the MyCAW assessment tool [37]. The HORIZONS team identified participants who highlighted pain as a problem on the QLQ-C30 pain subscale [38] using a cut-off of >25 (the clinically significant cut-off), cited pain on their case report form (CRF), consented to hear about further research and had not withdrawn from HORIZONS. The R.A.G.E chair shared information about the study in their newsletter and interested participants contacted the Chair.

Potential participants were sent a study introduction letter from the recruiting sites which explained about the study and why they had been approached. They also received a participant information sheet, a reply slip and a pre-paid return envelope addressed to the first author [JA]. The participant information sheet explained that all participants would need to be screened for eligibility prior to participating in the study. The reply slip included the option to decline participation in the study or provide contact details for further information. JA contacted interested potential participants by phone, discussed the study including the nature and purpose of the study, why they had been chosen, what the study would involve, and the advantages and risks of taking part. Potential participants were screened for eligibility and interviews were arranged. Verbal consent was taken at the time of interview and all interviews were recorded. The recruiting sites sent follow up letters to potential participants who had not responded to the first invitation.

### Data collection

Data were collected by semi-structured telephone interview at a single time point. All participants were offered the choice of a face-to-face interview, a video call or a telephone interview. The interviews were conducted by JA, a university lecturer with 20 years’ experience as a cancer nurse. A topic guide was developed prior to the interviews using recommendations for qualitative interviews [39]. The topic guide was reviewed and refined by two public contributors, who are cancer survivors living with chronic pain, to ensure the open questions would best elicit patients’ experiences. Participants were encouraged to share their story of their cancer diagnosis and treatment and were then asked about how things have been for them since. To gain rich information during the interview, participants were asked probing questions such as can you tell me more? can you describe? what do you think? How do you feel? can you reflect?

During the interviews, JA drew on her qualitative research and interview training, prior qualitative research experience, and her clinical experience of communicating with people with cancer.

After the interviews, participants were sent ‘thank you’ letters and a support leaflet. The leaflet contained contact details of the research team and advice about where to access support, such as national cancer charities. Interviews were digitally audio-recorded and transcribed verbatim by university-approved professional transcription services.

### Analysis

Transcribed interviews were imported into NVIVO Pro 12. JA familiarized herself with the data and generated initial codes. Multiple coders are not advocated in reflexive thematic analysis [2]. ‘Complete coding’ was undertaken whereby everything relevant to the research questions was identified from within the entire dataset [1, 3, 31]. Codes were then reviewed to construct themes. Themes were reviewed to ensure they captured the coded data extracts, had a central organising concept and reflected patterns across the dataset. Reflexive thematic analysis involves interpretation. Braun and Clarke (2022) explain that although analyses can be situated on a continuum from primarily descriptive, whereby researchers stay close to the data, to more interpretive analysis, there is interpretation across the spectrum. Thus, this experiential, critical realist analysis ’stayed close’ to the data and was nearer to the descriptive end of the spectrum, but was informed by the reflexive lens of the knowledge, experience and perceptions of the researcher, insights from theory and an understanding of the wider context [1]. Themes did not emerge from the data but were generated actively by JA reviewing, developing and rejecting candidate themes [1, 31, 40, 41]. Each theme encompassed a pattern of shared meaning organised around a central concept [1, 41].

### Impact of Covid-19 pandemic

During the study, there was a global pandemic of the Covid-19 virus. The original protocol gave participants the option of a telephone or face to face interview, however, due to the pandemic, the option for a face to face interview was removed. In real terms, this had minimal impact as the majority of interviews had taken place before the pandemic [74%, n = 14] and all participants had opted for a telephone interview. Thirty interviews had been planned but recruitment ceased after 19. Whilst this was partly related to the Covid-19 pandemic, the decision to end recruitment was predominately based on the high quality, richness and strength of the collected interview data. It was recognised in the protocol that a pre-determined sample size is not wholly congruent with the principles of qualitative research and sample sizes may change as a study develops [31, 42, 43].

### Reflexivity, rigour and trustworthiness

To support reflexivity, rigour and trustworthiness of the data analysis, and to enable a continuous process of critical thought and reflection, a reflexive journal was kept throughout the research. In addition, regular team meetings were used as an opportunity to critically discuss and reflect upon the research and findings. The public contributors were involved in the ‘member reflections’ [44] to determine if findings resonated with their experiences. This was an opportunity to engage in dialogue about the study’s findings and have opportunity to question, critique, feedback, affirm and collaborate with the researchers during the analysis. During this process the public contributors read interview transcripts and a summary of the findings produced by JA. They reflected on their own experiences and discussed with JA how their experiences aligned with the findings. The public contributors reported the findings mirrored their experiences and they recognised the issues raised by participants.

An audit trail of analysis was kept electronically. All coded data and lists of codes were stored within Nvivo. Electronic copies of all thematic maps developed throughout the analysis, including theme definitions, content of themes and candidate themes, have been saved.

## Results

### Sample

Across the four recruiting sites, 82 potential participants were invited to participate in the study and sent study information packs. 39 responded to the invitation (47.6%) of whom 20 agreed to be interviewed. During screening, one was identified as ineligible for the study. This was because they experienced long standing chronic pain that pre-dated their cancer treatment, resulting in 19 participants. Of the 19 who declined to be interviewed, the reasons cited were: they did not feel suitable (n = 11), they did not want to be interviewed (n = 5) or they had too many caring responsibilities [n = 2].

As shown in Table 1, the final sample (n = 19) consisted of 14 females and 5 males, with a mean age of 62.4 years at time of interview and a mean age of 46 years at diagnosis. Eight tumour groups were represented. Participants were between 18 months and 48 years since diagnosis. Over half (57.9%, n = 11) developed chronic pain within a year of cancer treatment ending and six participants [31.6%] developed chronic pain more than ten years after finishing cancer treatment.

**Table 1:** Sample characteristics (n = 19).

Demographic		Data
Gender	Male	5
	Female	14
Age at cancer diagnosis	Mean	46 years
	Range	19–74 years
Age at interview	Mean	62.4 years
	Range	38–78 years
Cancer	Breast	10
	Head and neck	2
	Head and neck and Non-Hodgkins Lymphoma	1
	Non-Hodgkins Lymphoma	1
	Endometrial	1
	Ovarian	1
	Testicular	1
	Hodgkins Disease	1
	Multiple myeloma	1
Time since end of cancer treatment	< 1 years	1
	1–5 years	5
	5–10 years	4
	>10 years	9
	Range	18 months—48 years
Time from end of cancer treatment to developing chronic pain	< 1 year	11
	1–5 years	1
	5–10 years	1
	>10 years	6

All interviews took place on the telephone and ranged from 43 to 86 minutes with a mean of 67 minutes. 14 interviews took place before Covid-19 (January and February 2020) and five within two months of the first UK lockdown (i.e. during March and April 2020).

#### Findings

Five themes were generated which highlighted the experience of chronic pain after cancer treatment for cancer survivors: ‘Hear me… believe me…. Please’, ‘Expectation versus reality’, ‘” don’t understand…. don’t understand”‘, ‘Negotiating the maze’ and ‘Validate my pain, validate me’.

*Hear me…*.*believe me…please*. This theme centred around the overwhelming sense that people living with chronic pain after cancer treatment did not feel listened to or heard by health care professionals. Participants expressed that, when they had tried to talk to health care professionals about their pain, they had not been listened to:

*“I don’t feel that I’ve been listened to …*.*it still wasn’t really addressed…*. *no-one would actually listen to the fact that I was still in a lot of pain” [Charlotte]*

And *‘finding somebody who wants to hear or wants to listen’ [Charles]* was a significant challenge for them. Furthermore, alongside not being listened to about their chronic pain, when participants tried to talk to health care professionals about it, at times, they felt healthcare professionals did not believe that chronic pain after cancer treatment was a genuine ailment:

*“There are a lot of health care professionals who don’t believe that half these things [chronic pain after cancer treatment] exist… I still bump into people [with chronic pain after cancer treatment] who’ve been told*, *by their GPs and doctors*, *‘just pull yourself together*. *there’s nothing wrong here*. *There’s nothing actually happening here’” [Thomas]*

*“And the consultant I saw at the pain clinic didn’t believe me …*. *He said*, *‘Oh*, *I don’t think you could have that*. *Not after all this time’” [Olivia]*

Participants expressed that they had needed to broach the subject of chronic pain after cancer treatment with health care professionals many times. This led to them having the impression that health care professionals were exasperated by them and, at times, this felt personal:

*“I have felt like I’m annoying* *, and that’s really hard” [Louise]*

*“I feel a pest if I go to the doctors’*, *because I don’t think they know what to do…*. *I’ve got a bit of dread …*. *they’ll think*, *‘Oh*, *my God*, *this bloke looks like a nightmare to deal with’” [William]*

Similarly, they thought health care professionals might have suspected they were making up how hard it was for them:

*“My GP is a very good GP* *, but I think he got to the point where he was almost thinking, ‘This is a hypochondriac’” [Thomas]*

*“I feel as if quite a few medical professionals have thought*, *‘Oh for god’s sake*. *This has got to be in the brain or something*. *Psychosomatic or whatever or attention seeking*, *or whatever you want to say’” [Fiona]*

When participants tried to highlight or discuss chronic pain after cancer treatment with health care professionals, they were often met with resistance:

*[when a participant asked the doctor if it could be chronic pain after cancer treatment] “he said*.. *‘You prove it to me and I’ll look into it’” [William]*

It seemed to participants that some health care professionals did not want to, or could not, make the connection between the pain that participants were experiencing and their previous cancer treatment:

“*I knew that the pain was there because of what I’d had in the past*, *but I don’t feel there was that correlation between the two… “[Charlotte]*

Some felt healthcare professionals ignored their chronic pain, dismissed their concerns and said *“Well*, *you know*, *you should be lucky*, *you’re still here*.*” [Charlotte]* or compared them to others with different side effects of cancer as Sarah explained *an ‘oncologist said*, *“Well you’re the lucky ones’* [to live and not have long term side effects to your continence or bowels] but she *‘didn’t feel lucky–I live in such pain*!*’*.

The sense that they were not listened to, or believed, left participants feeling both frustrated and desperate. Participants were frustrated for not being taken seriously and explained that they were *‘banging their head against the wall’ [Charlotte]*.

*Expectations versus reality*. This theme centred around the dichotomy of expectation of recovery after cancer treatment versus the reality of living in chronic pain after cancer treatment.

Many participants spoke at length about their cancer diagnosis and treatment and the challenges that time had brought, however, they also expressed that they had understood it was going to be a difficult time. Participants expected acute side effects, but they felt utterly unprepared for the risk of long-term effects. They had not anticipated having chronic pain after cancer treatment, or how hard their life would be. They struggled with making sense of a life-saving treatment having such negative and long-lasting consequences and found it challenging to comprehend ‘*I didn’t dream that by taking a cancer treatment to save my life would leave me in agony all the time*. *Just that seems ludicrous to give you something that’s going to make you feel like this” [Gillian]*.

Anticipated recovery after cancer was influenced by personal, cultural and societal factors arising from participants, their families, their social communities and the health care professionals they encountered. On a personal level, patients assumed and anticipated they would fully recover after cancer treatment. Participants expressed cultural expectations from themselves and their communities to behave, think and act in ‘positive’ ways and felt societal pressure to think positively. They explained that sometimes their friends ‘forgot’ about their chronic pain and would lean in to hug them, even if participants had explained that touch gave them shooting pains. They felt at times their friends and family were frustrated with them: *‘And my mum had been very supportive all through my cancer treatment*, *and as far as she was concerned it [cancer] had gone*. *So she wasn’t very understanding of me having this problem [chronic pain after cancer treatment] and just thought I’d get over it*, *I think’ [Olivia]*. Louise explained health care professionals had said she *‘should be over this by now’ [Louise]* and this was hard *‘I think the worst thing is when someone is telling me how I should feel or how I should be further along than I am*, *rather than just listening*. *I think that’s been the most awful thing’*.

The expectations of participants, and those around them, were contrary to the reality of living with chronic pain after cancer treatment. In reality, living with chronic pain after cancer treatment negatively impacted and shaped all aspects of participants’ daily lives: physical, emotional, social, sexual, spiritual and economic. William encapsulated this when saying *“I think it affects every aspect of my life*, *really*. *It’s who you are*. *It’s who I am*, *going into the world…I don’t think there’s any part that’s not touched by that”*. Participants expressed that living with chronic pain after cancer treatment was hard, relentless and felt endless. It felt as if their life had shrunk or diminished in some way:

*“I feel like my life has got smaller and smaller”* *[Sarah]*

And this really limited their life:

*“So many doors have shut to me, you know”* *[Sandra]*

This made them feel isolated, as expressed by Thomas when he said *“There were times when I felt completely isolated”* and by Olivia who said “*You just felt really alone”*.

The reality of living with chronic pain after cancer treatment was associated with loss. There was tangible loss of things participants used to do, such as hobbies, driving, or work. Sophie explained the impact of this as *“My hands can’t cope with doing too much and then I’m in extreme pain with my hands afterwards…*. *So*, *it does impact on my leisure time*, *the things I normally do to relax*, *which is annoying”*. Many participants had needed to stop working, and mourned the loss of their job:

*“I used to have a very physical job*. *I used to work outside*. *I was a gardener…*. *It was my life*, *it was my job; it was my everything” [Emily]*

Alongside loss of physical function, for many there was loss and change to their appearance. Harry articulated the impact of this when he said:

*“I’ve actually lost four inches in height… it’s psychologically so difficult to look at somebody in the chest when you used to look at them in the eye…* *you do feel a little bit inferior”*

Loss was also embodied in other aspects of their lives, for example financial loss and loss of enjoyment as Sandra explained that ‘*Everything that I’ve enjoyed*, *I’ve lost’*. Participants also discussed lost relationships:

*“It [chronic pain after cancer treatment] has totally*, *utterly ruined my life and my family’s and my marriage*. *Everything…*.. *I mean my relationship with my husband has I would say virtually broken down…*. *It’s [chronic pain after cancer treatment] destroyed a person*. *It’s destroyed a person’s marriage*. *It’s destroyed a person’s family*. *It’s destroyed a person’s friendship group” [Fiona]*

Furthermore, there was a lost sense of self, existential identity and who they are since living with chronic pain after cancer treatment:

“*You know*, *I*, *sort of*, *feel like I’ve lost my identity” [William]*. This resulted in loss of confidence, “*It’s like basically my confidence is shot–and by ‘confidence’ I mean physically*, *socially*, *professionally*, *sexually*, *and spiritually” [Louise]* and a loss of independence. Charlotte highlighted the impact of such loss, by explaining “*My whole life isn’t my own*. *That’s how I feel”*. For some, the loss of sense of self meant how they viewed themselves and the way they lived their lives was very unfamiliar to them and different from before. This was epitomised when Louise said *“I don’t recognise my life”* and this was at utter odds from the recovery they had expected after cancer. Many still lived with a sense of a Sword of Damocles over their heads regarding anxiety and fear relating to the risk of cancer recurrence:

*“That fear is horrendous*. *It’s still there*. *Five years down the line or six years down the line*, *I’m still… Every time I get a particular bad pain that suddenly appears from nowhere*, *you go down the same… Your brain takes you down that same road of*, *‘It’s back again*.*’” [Gillian]*

And the fear of cancer recurrence limited their enjoyment of day to day living:

*“You can’t get happy about anything because uhm you’re frightened in case the worst is going to happen if you know what I mean*?. ….*You don’t want to look forward to anything…It’s very emotional*. *You don’t want to build your hopes up too much” [Ben]*

This theme demonstrates the challenges surrounding the expectation of recovery after cancer treatment versus the reality of living in chronic pain after cancer treatment. Participants, and the perceptions of their families, social communities and health care professionals, had expected full recovery from cancer. Yet in reality, living with chronic pain after cancer treatment was really hard. It negatively impacted and shaped all aspects of participants’ daily lives: physical, emotional, social, sexual, spiritual and economic. It felt relentless and endless and embodied loss of function and sense of self. For many, reconciling those differences was difficult.

*Theme*: *‘They don’t understand…*. *We don’t understand’*. The central organising concept for this theme focused on chronic pain after cancer treatment being an unknown phenomenon. The lack of knowledge and understanding about chronic pain after cancer treatment threaded through all the interviews. Participants felt that they were not told about the risks, causes, symptoms or management of chronic pain after cancer treatment. Furthermore, it seemed that health care professionals did not have any knowledge about chronic pain after cancer treatment, nor understanding of the true impact that living with chronic pain after cancer treatment had on participants. There were two subthemes within this theme: ‘an unexpected experience’ and ‘grappling in the dark’

*An unexpected experience*. Participants felt unprepared for chronic pain after cancer treatment. Almost all participants felt they had not been told about the risk of chronic pain after cancer treatment at the time of cancer diagnosis or during their cancer treatment:

*“I don’t think I’d ever had one single conversation with anyone about pain at all…I started my chemo and nothing was ever said about pain at all” [Charlotte]*

However, the complexity about information recall was acknowledged as Nicole explained:

*“Well I may have been [told about the risk of chronic pain after cancer treatment] to be honest, but you know, when you’re going through all that, you don’t sort of uhm… I can’t honestly remember”*

Furthermore, because they had not been informed about chronic pain after cancer treatment, they did not know what to expect *“There was no mention of any permanent damage*. *So it was a bit of a shock really” [Olivia]* and thus, when symptoms started to appear, they did not understand them:

*“The arm was getting weaker all the time*, *which I didn’t understand…*. *I couldn’t understand it all” [Dawn]*

They also recognised that not understanding or expecting chronic pain after cancer treatment made it harder to manage and cope with:

*“That’s a difficult thing to manage, if you expect one thing and something else is happening” [Thomas]*

*“If you’re forewarned about something then I think you deal with it better*. *And I wasn’t informed of that*, *so I suppose I didn’t deal with it as well as I wanted to” [Olivia]*

Moreover, the lack of discussion about long term side effects, including chronic pain after cancer treatment, made it harder for them to come to terms with living with pain:

*“It’s [cancer treatment] taken away more than it should have done*, *and I was led to believe that it wouldn’t have taken away anything…*. *I grieve deeply about what I had and I’m finding it*, *as time goes on*, *I’m finding it harder and harder and harder to come to terms with what things are now” [Fiona]*

*Grappling in the dark*. Many participants were searching to understand chronic pain after cancer treatment. There was a strong sense some participants did not truly understand why they experienced chronic pain after cancer treatment, where it had come from, what caused it or why and *‘the lack of understanding of what’s happening to your body is real’ [Thomas]*.

There was a sense of desperation and exasperation from participants who were seeking explanation for the cause of their pain and how to manage it. Participants explained that ‘*nobody’s talked to you about it*, *you know*, *[pause] nobody’s ever said to me*, *“Oh this is happening because of whatever…… I don’t know*, *nobody’s ever told me*.*” [Sarah]*. The feeling of confusion and frustration was clear: *‘I would like the support from someone who knows and who’s truthful about what’s happening to my body*. *I want to know the facts*, *and that’s it [William]*. This frustration led to desperation to seek answers *because ‘no-one has any answers for me*, *or no-one properly investigate…*. *I don’t know*, *you know*, *I really*, *really don’t know…*..*’*.

This was exacerbated as they perceived health care professionals also had little understanding or knowledge. It seemed that health care professionals did not have any knowledge about chronic pain after cancer treatment, nor understanding of the true impact that living with chronic pain after cancer treatment had on participants. Many felt they had encountered health care professionals who “*didn’t know anything about it” [Olivia]* and this culminated in the feeling that *“They [health care professionals] don’t understand” [Fiona]*. Sarah ruefully expressed her frustration about health care professional lack of knowledge and understanding, and the impact this had on survivors’ experiences *‘we don’t understand… they don’t understand*!*’*.

This theme has highlighted the lack of knowledge and understanding about chronic pain after cancer treatment. Participants did not feel prepared or informed about the risk of chronic pain after cancer treatment and struggled to understand what chronic pain after cancer treatment was, why it had happened and how to manage it. Further, they felt health care professionals lacked knowledge about it and failed to understand the impact chronic pain after cancer treatment had on them.

*Negotiating the maze*. The central organising concept in this theme was that support for chronic pain after cancer treatment is hard to identify. Routes to support were messy and confusing and a lack of support started soon after cancer treatment had finished. Participants reported how they felt abandoned by acute cancer services at the end of treatment:

*“They basically say*, *‘Right*, *you’ve finished your treatment*. *Off you go*. *Goodbye…’ it is the feeling of being discarded” [Gillian]*

However, some also mentioned a similar feeling in relation to pain services, particularly if pain management interventions had been seemingly ineffective:

*“It’s been quite… I use the word ‘fleeting’*, *as in you’re sent to see someone for*, *like*, *six sessions*, *and then if you don’t seem to make the progress that they want*, *then that’s it*. *You’re back on your own*. *So*, *that’s quite hard really” [Charlotte]*

One participant summarised this by explaining that she had not felt *“accompanied” [Louise]* after her cancer diagnosis and subsequent diagnosis of chronic pain after cancer treatment.

When their chronic pain symptoms started, it was evident that participants found it difficult to identify and access services to help support them. Participants had to learn about chronic pain after cancer treatment themselves and seek their own support:

*“I had to go and search myself where I could get support…*. *it was quite a frustrating time*. *You felt you had to really stick by your guns and stick up for yourself…I just had to get on with it and just find out as much as I could” [Olivia]*

This was often because they did not feel they were provided with the information and support from health care professionals that they needed:

*“Then my problem was finding out what it was…*. *I did a load of investigation… because no doctor tells you there’s an option or there’s somebody you can talk to…*. *nobody is there to tell you at all” [Gillian]*

However, participants found this difficult to do and it took its toll on their wellbeing:

*“It’s been very time-consuming…It’s taken a huge amount of time and writing letters and emails and phoning people and trying to decide what to do and making decisions all on by yourself…*. *I had to think of all this stuff for myself” [Felicity]*

*“I do feel a bit like I was a floundering fish at times because I just didn’t know where to turn…*. *I didn’t know where to go for help …*. *I feel like I’ve had to go out and find things for myself*, *which hasn’t been easy with being in pain 24/7” [Charlotte]*

Furthermore, there was an apparent inequality regarding identifying support services. For example, some participants had only heard of specialist support because of connections in their personal life, such as playing golf with an oncology consultant, or having a neighbour whose daughter was a specialist nurse.

It was evident that some participants had needed to use their own personal drive and determination to identify specialist services and get referred to them, yet many participants complained that once they had been able to identify a support service, it was difficult to access it. This could be because of the delays involved with referral to a specialist support programme:

*“That took two years to get to [specialist support]*, *by the way–not easy*. *You need referrals…*.*It was a lot…*.. *I had to get a letter from my GP*, *blah*, *blah*, *blah*. *You have to jump through a lot of hoops” [Louise]*

Similarly, the challenge of having to travel to support not provided locally was reported:

*“That’s quite a long way to go for me*. *But unless you went online there isn’t anything local that you can go and join…that is a bit frustrating” [Olivia]*

Consequently, some people did not go, or ceased attending, because as Sophie explained *“It was just impractical”*. Furthermore, there were financial implications of travel and the costs of supportive treatment:

“You had to pay for it yourself … but unless you’ve got money to go to somebody, you can’t” [Sarah]

Many participants explained that they felt they had spent a lot of time bouncing between clinical services searching for support with their chronic pain after cancer treatment. William explained “*it was a bit like being a ping-pong ball… “*. They felt like there was conflict and confusion about whether their care needs fell under cancer services, pain services or primary care:

*“I can go to the [primary care] doctor and say, ‘I’m in pain’ but it’s not their area of expertise, you know, the GP… there’s no point going back to an oncology team, because they’re really busy” [Sophie]*

*“[I] actually ended up in the Pain Clinic uhm and uh the guy that I saw wasn’t too sure why I was there” [Nicole]*

It felt to participants that health care professionals did not know how to manage or relieve their chronic pain after cancer treatment and thus, simply referred them to another service:

*“I think, generally, people just want to put you onto somebody else, don’t they, really? Your GP wants you to go to the pain clinic, and the pain clinic try everything they can and then you’re back at your GP’s, aren’t you, really?” [Emily]*

Consequently, participants were trapped in a cycle of endless referral:

*“A lot of my life is spent waiting to go to different people*, *and then you go to the person*, *and they’ll say*, *‘Well*, *sorry*, *we can’t help you’*. *So*, *you can end up waiting six months and getting nowhere fast” [Charlotte]*

Participants felt unsure about where to go for help:

*“He [the GP] was telling me to go to talk to them at oncology about everything … They were telling me to go to him*, *basically*, *and I was there*, *stuck in the middle*, *not knowing what to do…*. *Basically*, *you’re stuffed because nobody really wants to know…” [Emily]*

This contributed to a sense of hopelessness regarding their pain and a belief that nothing can be done to help them:

*“There’s nothing they [doctors] can do*. *So*, *you know*, *I just think to myself*, *‘Well what’s the point of going*?*’” [Sarah]*

This theme has shown that participants found it hard to identify and access services to support them with their chronic pain after cancer treatment. They felt trapped in an endless cycle of referral whereby most healthcare professionals wanted to refer them on to someone else rather than help them. This resulted in participants feeling lost and alone.

*Validate my pain*, *validate me*. The power of a diagnosis of chronic pain after cancer treatment was the central organising concept for this theme. There was a lot of emotional turmoil for participants who had not had their pain adequately diagnosed, or the reasons behind it explained: *‘it’s like having a disability and not being able to satisfactorily diagnose it and have a way forward*, *you know*, *you’re*, *sort of*, *just- it’s like a ship with no sails or rudder*, *you’re just getting blown around’ [William]*

For participants who had received a diagnosis of chronic pain after cancer treatment, there was an overwhelming sense of relief. They took comfort in an actual diagnosis and relief in it being identified. They held an enormous value to the validation of their pain. This helped them manage their pain and is encapsulated by Louise:

*“They give me the name for it [chronic pain after cancer treatment]…*. *I just cried*. *It was like it was so amazing to have it understood that these particular kinds of pain associated with going through cancer were known and treatable in some ways*, *that they were not necessarily curable but that there were things that could help*. *It was amazing…*. *In a funny way*, *nothing’s changed*. *I still have those things*, *but the fact that I know I’m not crazy*, *and that I know that they happen*, *and I know that they’re common side effects from complex cancer [treatment]*, *it’s very reassuring*. *It’s very*, *very reassuring*. *It doesn’t technically make it less painful*, *but it*, *sort of*, *does*, *if that makes sense”*.

Many mentioned the relief at being diagnosed:

“[When I] was told I did have it [chronic pain after cancer treatment], that was a big relief” [Olivia]

Some explained that they had met health care professionals who did not appreciate the value or importance on the diagnosis of chronic pain after cancer treatment, as they thought a diagnosis would not make any clinical difference:

*“He [the GP] would say*, *‘Well*, *it may be [when asked if the pain was related to cancer]*, *but we can’t prove it*. *There’s no connection*. *Even if it was*, *what difference does it make now*?*’” [Thomas]*

And some participants speculated that health care professionals may not want to discuss or diagnose chronic pain after cancer treatment as they would then be attributing the pain to treatment they had prescribed, given or recommended:

*“They’re basically not really interested in taking–well, not responsibility, but you know what I mean” [Emily]*

*“None of them really want to put their name to it” [William]*

Having, the value of a chronic pain after cancer treatment diagnosis, and the subsequent validation of their experiences, should not be overestimated. It helped participants to manage and cope with the situation:

*“But now that I know this is just normal for this condition [chronic pain after cancer treatment]… I can cope*. *I think it was just having more enlightening about the whole thing about pain and about my particular type of pain*. *I think that was the greatest thing ever” [Dawn]*

Having their pain explained to enable them to understand it, made a difference to how they lived:

*I would say that I’m, on the whole, more positive…and I think that’s because I probably understand my pain a bit more’ [Nicole]*

The sense of relief at chronic pain after cancer treatment being identified and, most importantly, explained and believed, was palpable in interviews in those participants who had experienced it and as Dawn explained *‘it changed my life’*.

## Discussion

Almost 40% of people experience pain after curative cancer treatment has ended [11] and many report unmet need with regards to their pain [17–19] yet we know very little about cancer survivors’ lived experience of chronic pain after cancer treatment [21]. There is a growing body of evidence for the experience of living with chronic pain and over 20 reviews have been published in this area since 2012 [45]. However, most of the research into the experiences of chronic pain has been in the non-malignant population [21, 45]. From the limited prior evidence cancer survivors appear to experience physical and emotional difficulties, lack information, and consider their experience of chronic pain and cancer to be intrinsically interwoven [21]. The unique aspect of the pain experience for cancer survivors, whereby pain was viewed as an indicator of their current cancer status and a reminder of the threat that they had experienced to their mortality [18] justified an inductive approach to this research. This study presents new and important insight into these experiences. It shows that cancer survivors living with chronic pain after cancer treatment to do not feel heard, or listened to, when they tried to discuss their pain. The findings highlight the lack of information, knowledge and understanding about chronic pain after cancer treatment, among both cancer survivors and healthcare professionals. Participants felt that health care professionals did not have knowledge and understanding about chronic pain after cancer treatment, nor, for some, even believe it to be genuine. The non-malignant chronic pain literature also describes a sense of lost personal credibility when pain is not believed [20]. For participants in the current study, some experienced both a lack of belief that they were experiencing pain, but also, that the pain they experienced was related to their previous cancer treatment. Despite the negative impact of chronic pain on participants’ lives, data presented here shows participants encountered unclear and limited pathways for support and often bounced from one clinical team to another. Furthermore, identifying and accessing services was challenging, and the responsibility of this was often left to the cancer survivor. However, the benefits of encounters with knowledgeable and empathetic health care professionals were palpable and much relief was provided by a diagnosis of chronic pain after cancer treatment. Cancer survivor participants in this study, who had experienced being listened to, heard and believed by healthcare professionals felt valued. This acknowledgement led to a sense of relief and a feeling of empowerment and strength to go on. This is reflected in a recent meta-ethnography study synthesizing 195 qualitive studies to understand the process of healing for those living in chronic pain [45]. Toye et al. (2021) found that for people living in chronic pain, finding a voice and being heard by others was a key theme towards a healing journey. To feel that their suffering was given a voice, was understood, that they had time to tell their story, were believed and taken seriously, was lifechanging.

For many years, research, policy and clinical practice have advocated the importance of informed consent and it is a legal and ethical requirement to discuss side effects associated with treatments during the informed consent procedure [46]. Yet, in this study, no participant felt they had, or could remember being informed about the risk of chronic pain after cancer treatment. Information recall at a time of cancer diagnosis is known to be poor [47] and many cancer survivors report being unaware of their risk of late effects of cancer treatment [48]. Digesting information at diagnosis and early cancer treatment is complex, as there is a balance between the need for and the fear of information [49] however, it has long been recognised that people living with and beyond cancer have information needs about long term and late effects of cancer [50]. Individuals desire for knowledge for about cancer late effects varies [48] however, without knowledge of the risks of chronic pain, participants in the current study did not understand their symptoms when they started, and this made the experience of pain more anxiety provoking and harder to manage. In a global call to action Howell et al. (2021) [51] stressed the importance of preparing cancer patients and survivors for active involvement in their care; however, this is difficult if people are not equipped with knowledge regarding risks.

In the current study, not only was there a lack of information, but also, arguably more upsettingly for participants, a lack of understanding from heath care professionals was reported. Participants did not feel listened to or believed. Chronic pain literature highlights that not feeling believed, or heard, regarding the experience of living with chronic pain can lead people to feel invalidated and experience lost personal credibility [45]. At times, participants felt that health care professionals did not believe their chronic pain after cancer treatment really existed. Healthcare professionals can underestimate the prevalence, severity and impact of pain in cancer survivors [52]. Further research into health care professionals’ knowledge and understanding of chronic pain after cancer treatment is in progress by the authors of this paper.

There was an unclear and limited pathway for support for participants living with chronic pain after cancer treatment. Participants felt abandoned by health care professionals and felt that they were bounced between support services. Participants had difficulties identifying and accessing services to support them with their chronic pain after cancer treatment, and often they had to identify support services themselves or needed to have a personal connection to health care professionals. In the UK, there has been a seismic change to post-cancer follow-up over the past decade with the introduction of personalized stratified follow-up care and supported self-management [53, 54]. UK Policy has sought to improve communication between primary and secondary care and the NHS Long Term Plan [55] stresses the importance of creating genuine partnerships and engaging patients in decisions about their health and wellbeing. However, these ambitions have not yet been fully realized and there continue to be problems with communication and follow up within cancer services [10, 56]. This is a global challenge [51].

There is limited research regarding cancer survivors’ experiences of chronic pain after cancer treatment and that which is available only includes women [21]. Yet we know chronic pain can occur years after cancer treatment has ended and from many different cancer sites [11, 24]. This study is the first to include men and women. Despite actively seeking male participants, this study only included five men [approximately 25% of the total sample]. It can be challenging to recruit men to qualitative studies using semi structured interviews [57]. Men’s experiences have sometimes been overlooked when examining emotionally complex topics [58] and some researchers have reported a lack of emotional expression among male research participants as a challenge [58]. However, whilst the number of participants was small, their contribution was rich, and the male participants spoke freely and eloquently. The mean length of time for the male interviews was slightly shorter compared to the female interviews, however their responses were articulate, thoughtful and emotional. Both male and female participants expressed how living with chronic pain after cancer treatment shaped and impacted their lives physically, emotionally and socially. Gender did not appear to have a considerable impact on the reported experiences of living with chronic pain after cancer treatment, and the themes generated within the study were reflective of both male and female participants. Despite being offered a face-to-face interview, a video call or a telephone interview, all participants chose to have a telephone interview. Braun and Clarke (2013) acknowledge virtual interviews can be convenient and empowering for participants as they can be conducted from the comfort of their own home or in a location of their choosing. This may have been particularly apt for participants living with chronic pain.

This study is one of the first to include cancer survivors at later stages of survivorship. It is important to give voice to these participants because effects of cancer treatment can emerge years after treatment has concluded [24]. Interestingly, it did not appear that length of time living with chronic pain contributed to chronic pain acceptance and understanding. Rather than length of time, more influential was survivors’ perceptions of the information, support and interaction they had had with health care professionals. The quality of the interaction with health care professionals, whereby they felt informed of the risks of chronic pain, listened to, believed, and validated through a diagnosis of chronic pain after cancer treatment was key.

### Study limitations

Participants were limited to England thus they received their treatment predominately in the NHS health care system and this may limit the transferability of the findings to other countries. Survivors of childhood cancer were not included in the sample due to additional and unique concerns relating to the transition from paediatric to adult cancer services. However, it would be helpful to include childhood cancer survivors who experience chronic pain after cancer treatment in future research.

Previous research into the experience of cancer survivors living with chronic pain after cancer treatment has focused exclusively on breast cancer survivors [21]. Efforts were made during recruitment to this study to include participants with a multiplicity of different cancers and a mix of men and women, however, over half of the final sample included women with breast cancer. Reasons for this may include that people with breast cancer equate to the highest proportion of people living in chronic pain after cancer [11] and less than 1% of breast cancers are in men [59]. Furthermore, cancer survivors were required to self-identify as having chronic pain after cancer treatment to participate in this study. Gendered norms may have contributed as male patients can be seen as stoic and more likely to deny pain [60]. Therefore, male cancer survivors might not have considered this study relevant to them. Also, in this study, recruitment was strongest from centres who saw more women with breast cancer compared to other cancers or men. Future research should focus on recruitment sites that see more male cancer survivors.

## Conclusions

This study has demonstrated that living with chronic pain after cancer treatment has detrimental effects on many aspects of people’s lives. Survivors did not feel informed or prepared for the risk or reality of chronic pain after cancer treatment and this compounded the difficulties of coping with and managing their pain. Survivors felt that they had not been listened to when they tried to talk about their chronic pain after cancer treatment, nor at times, believed. They felt health care professionals lacked knowledge and understanding regarding chronic pain after cancer treatment. Survivors encountered unclear and limited pathways for support and often bounced from one support team to another. Identifying and accessing services was a challenge, and the responsibility of this was often left to the survivor. However, palpable relief and benefit was felt when health care professionals diagnosed and acknowledged chronic pain after cancer treatment.

To improve experiences of chronic pain after cancer, we need to prepare people living with and beyond cancer about the risks and symptoms of chronic pain after cancer. Furthermore, when people present with symptoms, they need to be heard, listened to, and believed. Further research is needed to better understand health care professionals’ knowledge, understanding and confidence to support people with chronic pain after cancer treatment. Health care professionals need to acknowledge and diagnose chronic pain after cancer treatment because, as this study has demonstrated, this can provide considerable support, relief and benefit to those affected.

## Supporting information

S1 File (PDF)

S2 File (PDF)
