# Supplementary material for: ‘I think it affects every aspect of my life, really’: Cancer survivors’ experience of living with chronic pain after curative cancer treatment in England, UK
Source: PLoS One. 2023 Sep 1;18(9):e0290967. doi: 10.1371/journal.pone.0290967 (PMC10473538; doi:10.1371/journal.pone.0290967)
Supplement: S1 File — (PDF) [file pone.0290967.s001.pdf]

A qualitative exploration of the experiences and needs of cancer survivors with chronic pain

This protocol has been written in accordance with the Health Research Authority (HRA) guidance for qualitative study protocols (HRA, 2018).

KEY STUDY CONTACTS

Chief Investigator	Julie Armoogum<br>Macmillan Senior Lecturer<br>University of the West of England<br>2B28, Glenside Campus<br>Blackberry Hill<br>Stapleton, Bristol, BS16 1DD
Sponsor	The University of the West of England<br>Glenside Campus<br>Blackberry Hill<br>Stapleton<br>Bristol<br>BS16 1DD
Funder(s)	Macmillan Cancer Support<br>The University of the West of England
Key Protocol Contributors	Prof Candy McCabe, University of the West of England, UK<br>Prof Diana Harcourt, University of the West of England, UK<br>Prof Claire Foster, University of Southampton, UK<br>Dr Alison Llewellyn, University of the West of England, UK

STUDY SUMMARY

The study will adopt an experiential qualitative design to explore the experience, needs and service provision for cancer survivors living with chronic pain, at different stages of the cancer care pathway. The research questions include:

- What is the experience of chronic pain for cancer survivors at different stages of the cancer care pathway?
- What are cancer survivors' views and experiences of services used to help them manage their pain?
- What support would they have liked to have received from healthcare services to help manage their pain?

A cross-section of up to 30 cancer survivors at different stages of survivorship who are living with chronic pain will be recruited from three centres in England and from the public via a social media campaign. Data will be collected via semi-structured interviews and will be analysed using Braun and Clarke's (2006) method of thematic analysis. Public Research Partners, who are cancer survivors living with chronic pain, have been involved with the development of the protocol. The study and findings will contribute to the submission of a doctoral thesis and will be disseminated via presentation at conferences and publication in academic journals. Participants will be sent a summary of the findings at the end of the study if they wish. The findings will also be disseminated to the public via charity websites and talks to local interested groups.

See figure 1 for an overview of the participant pathway through the study.

Figure 1: Participant pathway through the study

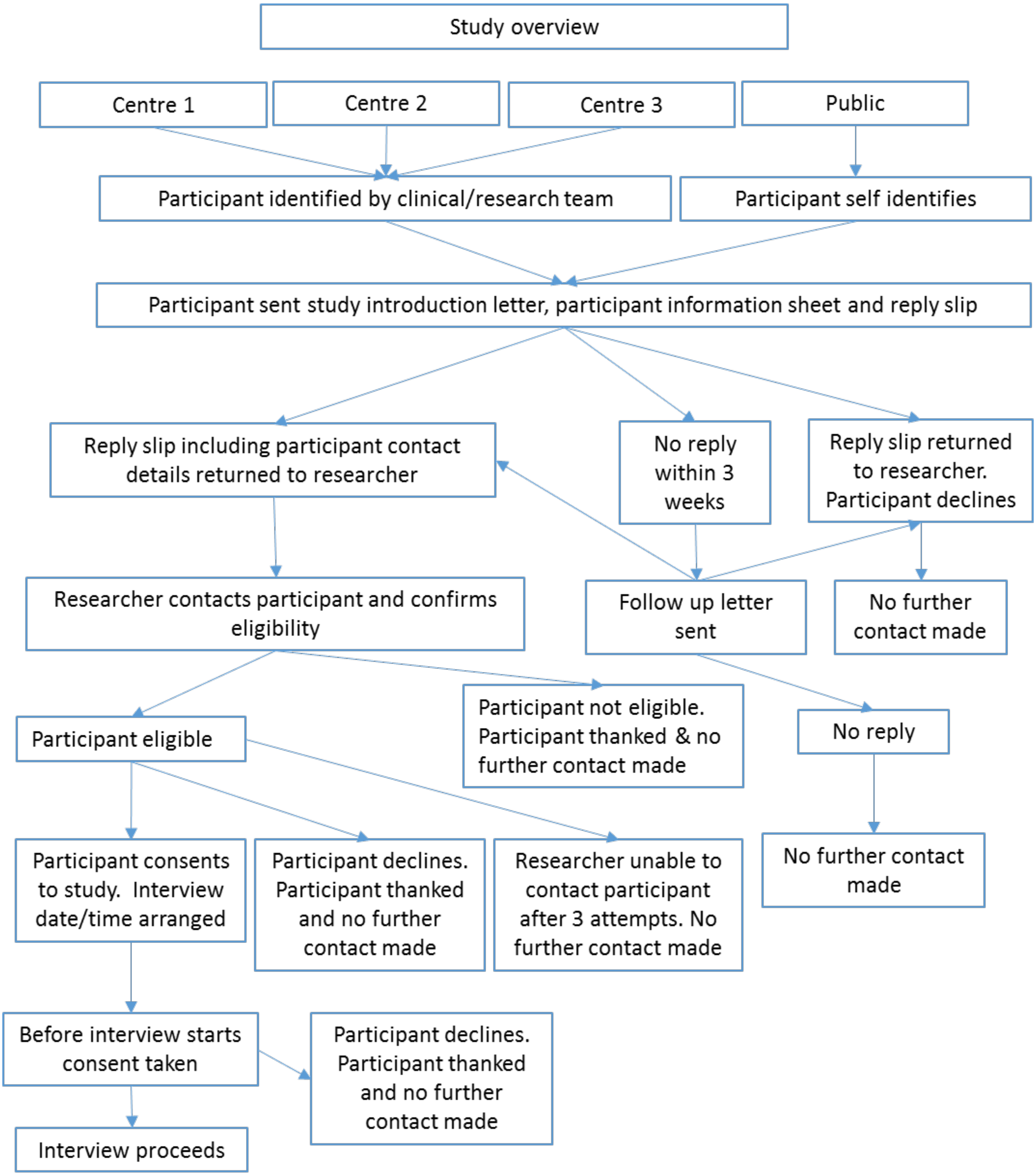

BACKGROUND

Survival rates for cancer are improving. More people are living for longer following their cancer treatment and it is predicted that by 2030, there will be 4 million people living with and beyond cancer in the UK (Maddams, Utley and Møller, 2012). However, people can experience many problems and symptoms in the months and years following treatment (Doyle, 2008; Armes *et al.*, 2009; Bennion and Molassiotis, 2013) and these problems can be difficult to manage (Maher *et al.*, 2018). Problems and symptoms can include severe fatigue, urinary problems such as incontinence and bowel dysfunction, anxiety, depression and chronic pain (Glare *et al.*, 2014; Maher *et al.*, 2018). Chronic pain can be a common problem for cancer survivors and a recent systematic review found that almost 40% of people diagnosed with cancer experience pain after treatment (Van Den Beuken-Van Everdingen *et al.*, 2016). Furthermore, almost a fifth of women living beyond a diagnosis of early stage breast cancer in the UK expressed an unmet need relating to pain (Capelan *et al.*, 2017). It is increasingly recognised that persistent and chronic pain is an under researched yet important area of concern. The National Cancer Research Institute announced in November 2018 that persistent pain is a top 10 research priority for those living with and beyond cancer. Work has been done to establish the risk factors associated with chronic pain after cancer treatment (Schreiber *et al.*, 2014; Bao *et al.*, 2018), however, to date, there is a paucity of research into the experiences and needs of adult cancer survivors with chronic pain and how they are supported.

A previous qualitative evidence synthesis, conducted as part of these doctoral studies, identified only four studies exploring the experiences of cancer survivors with chronic pain. All studies identified in the synthesis focused exclusively on women who had been diagnosed with breast cancer. Whilst it is challenging to draw conclusions when the evidence base is so limited, the synthesis suggested some important facets of the experience of chronic pain for cancer survivors. It highlighted that the experience of chronic pain and the experience of a diagnosis and treatment of cancer were interwoven and intricately related to each other. Chronic pain had a physical impact on daily lives and was an emotional experience. Cancer survivors reported that the chronic pain was an unexpected consequence of their treatment:

a consequence they had not anticipated living with and felt they were left to manage by themselves. Consequently, they adopted a variety of methods to help with managing their pain.

The proposed study aims to explore experiences and needs of cancer survivors with chronic pain, at different stages of the cancer care pathway, and to seek their views and experiences of service provision to help manage their chronic pain. This is an important area of study because it is known that chronic pain can be a problem for some cancer survivors (Van Den Beuken-Van Everdingen *et al.*, 2016). Currently, UK health policy focuses on those 'living with and beyond cancer' (Department of Health, 2017) and considers the needs of people affected by cancer collectively. However, whilst there may be some similarities in how chronic pain is experienced in those 'living with' cancer and those 'living beyond' cancer, there will undoubtedly be differences in the clinical services they have access to for support. During active treatment, people with cancer will be embedded in oncology services and should have access to support for symptoms, such as pain, during this time. Yet, research into the experience of cancer survivorship has identified that cancer survivors can feel 'dropped from the system' when treatment finishes as support from health care professionals can end abruptly at the end of treatment (Matthews and Semper, 2017). Thus it is important to capture the views of those 'living beyond' cancer as their experiences of chronic pain, and of services to support them, may be unique to this population. Research has highlighted that there are high unmet needs with regards to pain in this population (Capelan *et al.*, 2017) and thus it is essential to understand more about the experiences and needs of cancer survivors with chronic pain, and their experiences of service provision, to review how well current services meet their needs.

Operational terms:

Cancer survivor: This study will adopt the EORTC (European Organisation for the Research and Treatment of Cancer) definition that defines cancer survivor as any person diagnosed with cancer, who has completed his or her primary treatment (with the exception of maintenance therapy) who has no active disease (Moser and Meunier, 2014).

Chronic pain: Chronic pain that persists or recurs for longer than 3 months (IDC11).

Cancer care pathway: Five main phases of the cancer care pathway including: diagnosis and treatment (assumed to be a year from diagnosis), rehabilitation (assumed to be the year after treatment, estimated to be the second year since diagnosis), monitoring – split between early and late monitoring (includes those at risk of recurrence or treatment complications but with no active cancer or treatment related illness), progressive illness (includes incurable cancer but not those in the last year of life, and significant treatment illness) and end of life – (includes those in the last year of life, including those diagnosed within a year of death) (Maher and McConnell, 2011).
Services: Interactions between cancer survivors and healthcare professionals within the NHS or a third sector organisation e.g. follow up clinics, telephone support, specialist services, primary care, Macmillan health and wellbeing events.
Public research partner: People who are actively involved with the research design and process (Involve, 2019). For this research the Public Research Partners are cancer survivors living with chronic pain.

THEORETICAL FRAMEWORK

This study will adopt an experiential theoretical framework and will thus focus on participant standpoints and how they see the world (Braun and Clarke, 2013). It will take a critical realist approach and therefore adopt a theoretical perspective which assumes an ultimate reality but recognises that how reality is experienced and interpreted is shaped by culture, language and political interests (Braun and Clarke 2013, Green and Thorogood, 2018).

RESEARCH QUESTION/AIM(S)

Principal research objective

To explore the experience, needs and service provision for cancer survivors with chronic pain, at different stages of the cancer care pathway.

Secondary research questions

- What is the experience of chronic pain for cancer survivors at different stages of the cancer care pathway?
- What are cancer survivors’ views and experiences of services used to help them manage their pain?
- What support would they have liked to have received from healthcare services to help manage their pain?

DESIGN AND METHODOLOGY

The study will adopt an experiential qualitative design to explore the experience, needs and service provision for cancer survivors living with chronic pain, at different stages of the cancer care pathway.

Sample

Inclusion criteria

- Adults over 16 years old when diagnosed and treated for cancer in England
- People who self-report as having completed anti-cancer therapy (with the exclusion of hormone treatment) as per EORTC definition
- People with self-reported chronic pain that persists or recurs for longer than three months
- People able to communicate in the English language

Exclusion criteria

- Survivors of childhood cancer
- People with known active primary disease or metastatic disease
- People who are receiving active anti-cancer treatment
- People who have known terminal disease

- People previously seen by a chronic pain clinic team for non-malignant chronic pain

Sampling framework

This study will explore experiences of cancer survivors at different stages of the cancer care pathway, therefore, it is appropriate to purposively sample participants who are at different time points of their cancer pathway to answer this question. Based on Maher and McConnell's model of the cancer care pathway (Maher and McConnell, 2011), the time points will include:

- Rehabilitation (assumed to be the year after treatment, estimated to be the second year since diagnosis)
- Early monitoring 1 (up to 5 years from diagnosis)
- Early monitoring 2 (up to 10 years from diagnosis)
- Late monitoring (beyond 10 years from diagnosis)

Where possible, a mixture of men and women will be sampled with a variety of cancers.

Sampling centres

There will be four strands to recruitment and each centre will be targeting some or all of the time categories.

- Centre 1 - Referrals made to an NHS national Complex Cancer Late effects rehabilitation service (CCLERS) at the Royal National Hospital for Rheumatic Diseases (RNHRD), Bath. This is a late effects service therefore it is anticipated that this centre will identify potential participants for the 'up to 10 years' and 'over 10 years' categories. CCLERS sees men and women with any type of cancer, though the majority are post-breast cancer.

- Centre 2 - Self referrals made to a national cancer charity running support services for anyone affected by cancer (Penny Brohn UK). This service is open to men and women with any type of cancer and at any stage of the cancer pathway. It is anticipated potential participants will be identified for any of the time categories.
- Centre 3 - Participants from two national cohort studies exploring recovery of health and wellbeing following cancer treatment: ColoREctal Wellbeing cohort study (CREW) and Macmillan HORIZONS Programme run by the Macmillan Survivorship Research Group at the University of Southampton. CREW is a national study for men and women with colorectal cancer and opened in 2010. Potential participants will be suitable for the 'up to 5 years' and 'up to 10 years' time categories. As recruitment continues, either gender may be purposively sampled, to increase representation of men and women in the sample, if required. HORIZONS includes young women with breast cancer, women with gynaecological cancer and men and women with diffuse large B cell lymphoma. The study opened in September 2016 and it is anticipated potential participants will be identified for the 'rehabilitation' time category
- Public - A media campaign to recruit hidden populations (those who may not be engaged in the services or research above). This will include local radio, charities, local press, professional literature and magazines and professional networks. This may identify potential participants who are men or women, with any type of cancer, in any of the time categories.

Size of sample

Qualitative studies often have small sample sizes compared to quantitative studies (Braun and Clarke, 2013). This study will aim to recruit a maximum of 30 participants, with a representative spread of age, gender and time since treatment. This study will aim to recruit 5-8 people at each stage of the cancer care pathway (excluding diagnosis and treatment and end of life) proposed by Maher and McConnell, 2011).

Table 1: Proposed sample sizes for each stage of pathway and recruiting centres

Stage of cancer care pathway	Sample size	Centres (definitions on page 8)
Rehabilitation (assumed to be the year after treatment, estimated to be the second year since diagnosis)	5-8	2, 3 and public
Early monitoring 1 (up to 5 years from diagnosis)	5-8	2, 3 and public
Early monitoring 2 (up to 10 years from diagnosis)	5-8	1, 2, 3 and public
Late monitoring (beyond 10 years from diagnosis)	5-8	1, 2 and public

The sample size of up to 30 individual interviews was decided upon based on discussions with the supervisory team and the recommendations of sample sizes for studies using thematic analysis (Braun & Clarke, 2018, from the "frequently asked questions" section of website) and for studies which aim to identify patterns across data (Terry & Braun, 2011). Furthermore, Mason (2010) suggests up to 30 interviews will generate sufficient data for doctoral study using qualitative methodology. However, it is recognised that a pre-meditated approach to the sample size is not wholly congruent with the principles of qualitative research (Mason 2010, Braun & Clarke 2013) and it may change as the study develops if data saturation is reached (Green & Thorogood, 2018). Saturation is used in qualitative research as a criterion for discontinuing data collection or analysis (Saunders *et al.*, 2018). This study will adopt Saunders et al (2018) definition of data saturation as ''the degree to which new data repeat what was expressed in the previous data' (p.1897) and thus data collection will cease when this occurs.[1]

[1] Saturation is a concept arising from grounded theory, whereby new data are not adding to the emerging theory and thus theoretical saturation is reached (Glaser & Strauss 1967, Green & Thorogood 2018). It can be a contentious concept and whilst it is seen by some as the 'gold standard' (Guest, Bunce and Johnson, 2006), others argue that some authors claim to have reached saturation whilst providing little evidence of the density of theory required if a grounded theory approach had been taken (Green & Thorogood, 2018). There is increasing recognition of need to clarify the difference between 'theoretical saturation' and 'data saturation' and Saunders et al (2018) have recently proposed defined theoretical saturation as 'the

RECRUITMENT

Participant identification – Centres 1, 2 and 3

Identification of participants is specific to each centre depending on their referral procedures and standard measurements for pain.

Centre 1 – Centre team will review referral lists for CCLERS in past two years to identify potential participants.
Centre 2 – Centre team will review registration forms of clients and identify clients who highlighted pain as a concern in the MyCAW assessment tool.
Centre 3 – Centre team will identify participants who highlighted pain as a problem on the QLQ-C30 pain subscale, using a cutoff of >25 (the clinically significant cutoff), and who have consented to hear about further research and have not withdrawn from CREW/HORIZONS.

Potential research participants who are referred to centres 1 and 2 after the study has opened will be identified by centre teams on referral/self-referral.

Participant identification – public recruitment

The study will be advertised to members of the public via local radio, press, social media and dissemination through professional networks. Julie Armoogum's (JA) contact details will be given. People who are interested in participating will contact JA via email or telephone.

Screening of participants - Centres 1, 2 and 3

development of theoretical categories: related to the grounded theory methodology' and data saturation as ''the degree to which new data repeat what was expressed in the previous data' and thus focuses on the data collection period (p.1897).

JA will provide the centre teams with a screening log with predetermined study numbers (Appendix A). Participants will be identified by the centre teams as described above and the centre teams will enter participants on the screening log. The centre team will send participants a study pack including:

1) A study introduction letter from the clinical/research team on centre headed paper (Appendix B)
2) A Patient Information Sheet (Appendix C)
3) A reply slip (Appendix D)
4) Pre-paid return envelope

The reply slip and consent form will be labelled with the participant's study number. The centre team will email JA with the study numbers when the study packs have been sent. JA will contact the centre team three weeks later to request a follow up letter (Appendix F) to be sent to any participants who have not responded.

Potential research participants who are referred to centres 1 and 2 after the study has opened will be identified by centre teams on referral/self-referral. The procedure will follow as outlined above.

Screening of participants – public recruitment

JA will enter participant details on screening log with predetermined study numbers (Appendix A). JA will send participants a study pack and follow the procedures as detailed above.

Consent

After the participant has received the study pack and returned the reply slip, JA will contact the participant via the telephone number provided. During this call JA will describe the study in more detail, confirm eligibility and answer any questions participants may have. To confirm eligibility, JA will complete an eligibility screen (Appendix G). If participants are eligible and would like to participate then a mutually agreeable time will be arranged for the interview.

Consent will be taken at the time of interview (Appendix E). For participants having a face-to-face interview, written consent will be secured. For participants having a telephone or Skype interview, verbal consent will be audio recorded. Each of the statements on the consent form will be read to participants and their responses will be audio recorded.

Participants will be asked if they give permission for their General Practitioner (GP) to be informed of their involvement in the study. If so, a letter will be sent to the GP (Appendix H).

DATA COLLECTION

Data will be collected by semi-structured interview at a single time point. Interviews will either be face-to-face or over the telephone or Skype based on participant preference and practicalities of travel. Face to face interviews will be conducted either at the University of the West of England, Penny Brohn UK or Royal United Hospitals Bath. Refreshments will be provided. It is anticipated that interviews will last between one and two hours but will continue until the interviewee has finished. An interview schedule is available in Appendix I. The interviews will be conducted by JA. JA will draw on her qualitative research training, her prior qualitative research experience, and her clinical experience of communicating with people with cancer during the interviews. When the interview has finished, participants will be given information about charities who can offer support and information (Appendix J). JA has undertaken interview training and has conducted a series of practice semi-structured interviews with people affected by cancer. Interviews will be digitally audio-recorded. Recorded audio interviews will be orthographically transcribed by UWE transcription services. Participants can have a copy of their interview transcript if they wish. Transcripts will follow the Braun and Clarke (2013) transcription notation system. After the first three interviews, JA and supervisory team will review the transcripts and modify the interview schedule as required.

ANALYSIS

Transcribed interviews will be imported into NVIVO Pro (QSR International, 2016). Interview data will be analysed using experiential thematic analysis (Braun and Clarke, 2013). The first phase of analysis will involve JA familiarizing herself with the data. Audio recordings will be listened and re-listened to and transcriptions will be read and re-read and initial ideas about the data will be noted. This is not a passive process but one whereby words are read and listened to actively, analytically and critically (Braun and Clarke, 2006). The second phase involves generating initial codes. A process of 'complete coding' (Braun and Clarke, 2006 p.206) will take place whereby everything relevant to the research questions will be identified within the entire dataset. Semantic codes will be applied to capture surface meaning of the data and latent codes will be used to capture implicit meanings, assumptions and theoretical frameworks underpinning the data. Codes will then be reviewed and organised to generate themes. All the coded data relevant to each theme will be gathered together within each generated theme. Themes will then be reviewed to ensure they capture the coded data extracts and a thematic map will be created. The process of defining and naming themes will continue by ongoing analysis to refine the specifics of each theme. Finally, a report will be produced, including extract examples and final analysis of selected extracts and relating the analysis to the research question and literature (Braun and Clarke, 2006, 2013).

Some authors highlight that having multiple coders can increase reliability of the analysis (Green & Thorogood, 2018) yet Braun and Clarke (2018, from website) suggest that there is no one accurate way to code data as coding is an active and reflexive process that reflects the researcher. Thus, multiple coders, and inter-relater reliability, serves to demonstrate that researchers have been trained to code the same way, rather than improving the accuracy of the coding (Braun and Clarke, 2018). However, use of a single coder assumes a level of skill of the researcher. As JA is a novice researcher, a detailed report, including sections of transcripts, will be presented to the supervisory team to discuss how themes have been generated.

PATIENT AND PUBLIC INVOLVEMENT

Public Research Partners have been involved with the design of this research. Public Research Partners were recruited by:

- Discussion with health care professionals to see if they could approach anyone who may be suitable
- Dissemination via the South West Cancer Alliance Cancer Operations Group and Somerset, Wiltshire, Avon and Gloucestershire (SWAG) Cancer Alliance Macmillan Patient and Public Engagement Lead
- Advertisement of the opportunity on cancer and research websites including Jos Trust and the NIHR People in Research websites
- Use of twitter to promote the posts on the websites above

Public Research Partners reviewed the protocol and all participant study documentation. Any comments or suggestions were considered, discussed with the Public Research Partners and supervisory team, and incorporated into the final versions where possible.

ETHICAL AND REGULATORY CONSIDERATIONS

Assessment and management of risk

A full risk analysis has been conducted and a UWE risk assessment has been completed and is currently being verified by the university. A UWE Research Governance Record will be maintained throughout the study.

Research Ethics Committee (REC) and other Regulatory review & reports

Ethical approval from an NHS Research Ethics Committee (NHS REC) and UWE Research Ethics Committee will be sought prior to the study commencing. Substantial amendments that require review by NHS REC will not be implemented until approval has been granted and other mechanisms are in place to implement at site. All correspondence with the REC will be retained by JA. An annual progress report (APR) will be submitted to the REC within 30 days of the anniversary date on which the favourable opinion was given, and annually until the

study is declared ended. If the study is ended prematurely, JA will notify the REC, including the reasons for the premature termination. Within one year after the end of the study, JA will submit a final report with the results, including any publications/abstracts, to the REC.

Regulatory Review & Compliance

Before any site can enrol patients into the study, the Chief Investigator will ensure that appropriate approvals from participating organisations are in place.

PEER REVIEW

In addition to the key contributors to the protocol, the protocol has been peer reviewed by two independent experts who have knowledge of the relevant discipline to consider the clinical and/or service based aspects of the protocol.

DATA PROTECTION AND PATIENT CONFIDENTIALITY

All data generated by the study will be pseudonymised and any identifying information will be replaced by an unrelated sequence of characters known as the study number. During the interview recordings, no personal identification information will be recorded and all participants will be referred to by study numbers only in any publications resulting from the study.

A Data Management Plan has been created for managing data generated in this study. Any personal information obtained or data generated during the study will be securely kept on UWE password protected documents and stored on UWE password protected OneDrive cloud storage. This will be kept separately from interview data and will be destroyed when no longer required. Signed copies of the informed consent forms will be scanned on a UWE owned and managed scanner and stored in a password protected folder on a UWE password protected OneDrive cloud storage facility. Paper copies will then be securely destroyed.

No personal data will be shared. Pseudonymised interview data will be shared between the research team within UWE password protected systems. One member of the team is employed by the University of Southampton. If required, a Data Sharing Agreement will be in place between UWE and the University of Southampton before any data are shared. Any data to be shared with others outside of the research team will be covered by a Data Processing Agreement (for example, transcribers).

The Participant information sheets contain a GDPR transparency statement as recommended by the Health Research Authority and include a UWE Privacy Notice outlining how UWE collects, manages and uses personal data before, during and after participation in the research. At the end of the study, data will be archived in a UWE research data archive facility and kept for eight years.

INDEMNITY

The study is sponsored by University of the West of England, Bristol and is covered by the universities Professional Indemnity Insurance.

DISSEMINATION POLICY

The study and findings will contribute to the submission of a doctoral thesis and will be disseminated via presentation at conferences and publication in academic journals. Participants will be sent a summary of the findings at the end of the study if they wish. The findings will be disseminated to the public via charity websites and talks to local interested groups.

Appendix A – Screening logs: 1) For centre 1 2) For centre 2 3) For centre 3 4) For Public

Centre 1: Complex Cancer Late Effects Rehabilitation Service (CLERS) at the Royal National Hospital for Rheumatic Diseases (RNHRD)

**A qualitative exploration of the experiences and needs of cancer survivors with chronic pain**				
Centre 1: Complex Cancer Late Effects Rehabilitation Service (CLERS) at the Royal National Hospital for Rheumatic Diseases (RNHRD)				
Study number	Patient name	Hospital number	Date study pack sent	Date follow up letter sent
CLERS1				
CLERS2				
CLERS3				
CLERS4				
CLERS5				
CLERS6				
CLERS7				
CLERS8				
CLERS9				
CLERS10				
CLERS11				
CLERS12				
CLERS13				
CLERS14				
CLERS15				
CLERS16				
CLERS17				
CLERS18				
CLERS19				
CLERS20				

Centre 2: Penny Brohn UK

**A qualitative exploration of the experiences and needs of cancer survivors with chronic pain**				
Centre 2: Penny Brohn UK				
Study number	Patient name	Centre number	Date study pack sent	Date follow up letter sent
PB1				
PB2				
PB3				
PB4				
PB5				
PB6				
PB7				
PB8				
PB9				
PB10				
PB11				
PB12				
PB13				
PB14				
PB15				
PB16				
PB17				
PB18				
PB19				
PB20				

Centre 3: ColoREctal Wellbeing cohort study, University of Southampton

A qualitative exploration of the experiences and needs of cancer survivors with chronic pain				
Centre 3: ColoREctal Wellbeing cohort study, University of Southampton				
Study number	Patient name	CREW study number	Date study pack sent	Date follow up letter sent
CREW1				
CREW2				
CREW3				
CREW4				
CREW5				
CREW6				
CREW7				
CREW8				
CREW9				
CREW10				
CREW11				
CREW12				
CREW13				
CREW14				
CREW15				
CREW16				
CREW17				
CREW18				
CREW19				
CREW20				

Centre 3: HORIZONS study, University of Southampton

**A qualitative exploration of the experiences and needs of cancer survivors with chronic pain**				
Centre 3: HORIZONS study, University of Southampton				
Study number	Patient name	HORIZONS study number	Date study pack sent	Date follow up letter sent
HOR1				
HOR2				
HOR3				
HOR4				
HOR5				
HOR6				
HOR7				
HOR8				
HOR9				
HOR10				
HOR11				
HOR12				
HOR13				
HOR14				
HOR15				
HOR16				
HOR17				
HOR18				
HOR19				
HOR20				

4. Public

A qualitative exploration of the experiences and needs of cancer survivors with chronic pain			
Public recruitment			
Study number	Participant name	Date study pack sent	Date follow up letter sent
PUB1			
PUB2			
PUB3			
PUB4			
PUB5			
PUB6			
PUB7			
PUB8			
PUB9			
PUB10			
PUB11			
PUB12			
PUB13			
PUB14			
PUB15			
PUB16			
PUB17			
PUB18			
PUB19			
PUB20			

Appendix B – **Study introduction letters:** 1) For centre 1 2) For centre 2 3) For centre 3 and 4) for participants who responded to the social media campaign.

1) RNHRD headed paper

Dear……

We are writing to you to let you know about a research study that may be of interest to you. This is because you have been referred to the Complex Cancer Late Effects Rehabilitation Service (CLERS) at the Royal National Hospital for Rheumatic Diseases (RNHRD) in Bath.

A team of researchers at the University of the West of England in Bristol are exploring the experiences and needs of cancer survivors with chronic pain. In this study, researchers are particularly interested in the experiences of people who have completed their cancer treatment as an adult and their cancer has gone, but who are living with chronic pain as a consequence of their cancer treatment. You may feel that you would like to be part of this research.

The research study fits into a programme of work that aims to establish healthcare needs of cancer survivors with chronic pain and review how well current services meet their needs. This will be submitted as a doctoral thesis by Julie Armoogum, Macmillan Senior Lecturer, University of the West of England. The PhD supervisory team includes Diana Harcourt (University of the West of England, Bristol), Professor Claire Foster (University of Southampton), Dr Alison Llewellyn (University of the West of England) and myself.

We have enclosed further details about the study, including an information sheet, a reply slip and a self addressed prepaid envelope. If you would like further information, please complete and return the reply slip. Julie Armoogum will call you to discuss the study in more detail.

If you would like more information about the research, please do not hesitate to contact Julie Armoogum:

Julie Armoogum, Macmillan Senior Lecturer, University of the West of England, 2B28, Glenside Campus, Blackberry Hill, Bristol, BS16 1DD

Telephone: 0117 32 88658 Email: Julie.armoogum@uwe.ac.uk

With very best wishes,

Professor Candy McCabe

Professor Candy McCabe, Florence Nightingale Foundation Clinical Professor in Nursing, University of the West of England, Bristol & Royal United Hospitals NHS Foundation Trust, Royal National Hospital for Rheumatic Diseases, Upper Borough Walls, Bath BA1 1RL

Personal Assistant: Lindsay Davies. Telephone: 01225 473479 Email: lindsay.davies2@nhs.net

2. Penny Brohn headed paper

Dear……

We are writing to you to let you know about a research study that may be of interest to you. This is because when you visited Penny Brohn UK you listed pain as one of your concerns.

A team of researchers at the University of the West of England in Bristol are exploring the experiences and needs of cancer survivors with chronic pain. In this study, researchers are particularly interested in the experiences of people who have completed their cancer treatment as an adult and their cancer has gone, but who are living with chronic pain as a consequence of their cancer treatment. You may feel that you would like to be part of this research.

The research study fits into a programme of work that aims to establish healthcare needs of cancer survivors with chronic pain and review how well current services meet their needs. This will be submitted as a doctoral thesis by Julie Armoogum, Macmillan Senior Lecturer, University of the West of England. The PhD supervisory team includes Professor Candy McCabe (University of the West of England, Bristol), Professor Diana Harcourt (University of the West of England, Bristol), Professor Claire Foster (University of Southampton) and Dr Alison Llewellyn (University of the West of England).

We have enclosed further details about the study, including an information sheet, a reply slip and a self addressed prepaid envelope. If you would like further information, please complete and return the reply slip. Julie Armoogum will call you to discuss the study in more detail.

If you would like more information about the research, please do not hesitate to contact Julie Armoogum:

Julie Armoogum, Macmillan Senior Lecturer, University of the West of England, 2B28, Glenside Campus, Blackberry Hill, Bristol, BS16 1DD

Telephone: 0117 32 88658 Email: Julie.armoogum@uwe.ac.uk

With very best wishes,

Dr Marian Naidoo

Director of Services, Penny Brohn UK

3. Southampton headed paper - CREW

Dear……

We are writing to you to let you know about a research study that may be of interest to you. This is because you are part of a cohort study to explore recovery of health and wellbeing following primary treatment of colorectal cancer (ColoREctal Wellbeing cohort study) run by the Macmillan Survivorship Research Group in Southampton. In your last questionnaire for the study, you listed pain as a problem.

A team of researchers at the University of the West of England in Bristol are exploring the experiences and needs of cancer survivors with chronic pain. In this study, researchers are particularly interested in the experiences of people who have completed their cancer treatment as an adult and their cancer has gone, but who are living with chronic pain as a consequence of their cancer treatment. You may feel that you would like to be part of this research.

The research study fits into a programme of work that aims to establish healthcare needs of cancer survivors with chronic pain and review how well current services meet their needs. This will be submitted as a doctoral thesis by Julie Armoogum, Macmillan Senior Lecturer, University of the West of England. The PhD supervisory team includes Professor Candy McCabe (University of the West of England, Bristol), Professor Diana Harcourt (University of the West of England, Bristol), Dr Alison Llewellyn (University of the West of England) and myself.

We have enclosed further details about the study, including an information sheet, a reply slip and a self addressed prepaid envelope. If you would like further information, please complete and return the reply slip. Julie Armoogum will call you to discuss the study in more detail.

If you would like more information about the research, please do not hesitate to contact Julie Armoogum:

Julie Armoogum, Macmillan Senior Lecturer, University of the West of England, 2B28, Glenside Campus, Blackberry Hill, Bristol, BS16 1DD

Telephone: 0117 32 88658 Email: Julie.armoogum@uwe.ac.uk

With very best wishes,

Professor Claire Foster

Professor of Psychosocial Oncology, Director of Macmillan Survivorship Research Group

University of Southampton

Southampton headed paper - HORIZONS

Dear……

We are writing to you to let you know about a research study that may be of interest to you. This is because you are part of the HORIZONS study run by the Macmillan Survivorship Research Group in Southampton. In your last questionnaire for the study, you listed pain as a problem.

A team of researchers at the University of the West of England in Bristol are exploring the experiences and needs of cancer survivors with chronic pain. In this study, researchers are particularly interested in the experiences of people who have completed their cancer treatment as an adult and their cancer has gone, but who are living with chronic pain as a consequence of their cancer treatment. You may feel that you would like to be part of this research.

The research study fits into a programme of work that aims to establish healthcare needs of cancer survivors with chronic pain and review how well current services meet their needs. This will be submitted as a doctoral thesis by Julie Armoogum, Macmillan Senior Lecturer, University of the West of England. The PhD supervisory team includes Professor Candy McCabe (University of the West of England, Bristol), Professor Diana Harcourt (University of the West of England, Bristol), Dr Alison Llewellyn (University of the West of England) and myself.

We have enclosed further details about the study, including an information sheet, a reply slip and a self addressed prepaid envelope. If you would like further information, please complete and return the reply slip. Julie Armoogum will call you to discuss the study in more detail.

If you would like more information about the research, please do not hesitate to contact Julie Armoogum:

Julie Armoogum, Macmillan Senior Lecturer, University of the West of England, 2B28, Glenside Campus, Blackberry Hill, Bristol, BS16 1DD

Telephone: 0117 32 88658 Email: Julie.armoogum@uwe.ac.uk

With very best wishes,

Professor Claire Foster

Professor of Psychosocial Oncology, Director of Macmillan Survivorship Research Group

University of Southampton

4. UWE Bristol headed paper

Dear……

Thank you for your recent interest in participating in a research study exploring the experiences and needs of cancer survivors with chronic pain.

In this study, we are particularly interested in the experiences of people who have completed their cancer treatment as an adult and their cancer has gone, but who are living with chronic pain as a consequence of their cancer treatment. You may feel that you would like to be part of this research.

Please find enclosed further details about the study, including an information sheet, a reply slip and a self addressed prepaid envelope. If you would like further information, please complete and return the reply slip.

The research study fits into a programme of work that aims to establish healthcare needs of cancer survivors with chronic pain and review how well current services meet their needs. The work will be submitted as a doctoral thesis and the PhD supervisory team includes Professor Candy McCabe (University of the West of England, Bristol), Professor Diana Harcourt (University of the West of England, Bristol), Professor Claire Foster (University of Southampton) and Dr Alison Llewellyn (University of the West of England, Bristol).

We have enclosed further details about the study, including an information sheet, a reply slip and a self addressed prepaid envelope. If you would like further information, please complete and return the reply slip. I, Julie Armoogum, will call you to discuss the study in more detail.

If you would like more information about the research, please do not hesitate to contact me:

Julie Armoogum, Macmillan Senior Lecturer, University of the West of England, 2B28, Glenside Campus, Blackberry Hill, Bristol, BS16 1DD

Telephone: 0117 32 88658 Email: Julie.armoogum@uwe.ac.uk

With very best wishes,

Julie Armoogum

Macmillan Senior Lecturer

University of the West of England

Appendix C: Participant Information Sheet

UWE headed paper

Participant Information Sheet: An exploration of the experiences and needs of cancer survivors with chronic pain.

We would like to invite you to take part in our research study. Joining the study is entirely up to you. Before you decide it is important for you to understand why the research is being done and what it will involve. Please take time to read the following information carefully and discuss it with others if you wish. Ask us if there is anything that is not clear or if you would like more information. Take time to decide whether or not you wish to take part. Thank you for reading this.

What is the nature and purpose of this research?

This research looks at chronic pain in people who have had cancer. We are particularly interested in the experiences of people who have completed their cancer treatment as an adult and their cancer has gone, but who are living with chronic pain as a consequence of their cancer treatment. We will use the term 'cancer survivor' to describe who we would like to participate in the research.

We know that chronic pain can be a problem for some cancer survivors. However, little is known about what it is like to live with chronic pain as a cancer survivor and how people are supported by healthcare services to help manage their pain.

The research aims to explore the experience, needs and service provision for people who have had cancer and are living with chronic pain. It seeks to answer:

- What is the experience of chronic pain for cancer survivors at different stages of the cancer care pathway?

- What are cancer survivors' views and experiences of services used to help them manage of their pain?
- What support would they have liked to have received from healthcare services to help manage their pain?

These questions will be explored by interviewing cancer survivors who live with chronic pain.

The Chief Investigator for the study is Julie Armoogum, Macmillan Senior Lecturer at the University of the West of England in Bristol. This research will be part of a programme of work that will be submitted as a doctoral thesis.

Why have I been chosen?

You have been chosen to be considered for this study because you have had cancer treatment and your cancer has gone but you are living with chronic pain as a consequence of your treatment.

What would taking part involve?

Firstly, you would give your contact details to Julie Armoogum by returning the enclosed reply slip. Julie Armoogum will contact you to discuss the study in more detail, confirm your eligibility and answer any questions you may have. If you choose to take part in the research, we will then arrange a mutually convenient time for you to talk to Julie Armoogum about your experiences of living with chronic pain as a cancer survivor. The interview can be either face-to-face or over the telephone or Skype, depending on your preference and practicalities of travel. Face-to-face interviews can be at the University of the West of England in Bristol, Penny Brohn UK in Bristol or the Royal United Hospital in Bath. Travel expenses and refreshments will be available if the interview is face-to-face. Julie Armoogum will phone you if you decide on the phone option. It is thought the interviews will take between one and two hours, but will continue as long or as little as you would like. The interviews will be audio

recorded and then turned into written script. You can have a copy of the written script if you wish.

We would ask your permission to inform your GP about your involvement in the study.

We may also ask your permission for you to be contacted at a later date to consider participating in future research relating to this programme of doctoral studies.

Do I have to take part?

Taking part in the research is entirely voluntary. It is up to you to decide whether or not to take part. If you do decide to take part you will be given this information sheet to keep and be asked to give a consent. If you decide not to take part, the care you receive will not be affected.

What are the possible benefits of taking part?

There will be no direct benefit to your care or treatment by taking part in this research. However, you will be helping to inform clinical practice and services for the future.

What are the possible disadvantages and risks of taking part?

Thinking about, remembering and reflecting on your experiences may be upsetting. Care will be taken to ensure the interview is as supportive for you as possible and information will be given about charities who can offer further support and information.

What if something goes wrong?

If you are concerned or worried about any aspect of this research please contact Julie Armoogum in the first instance. You can also contact the Director of Studies, Professor Candy McCabe (contact details are at the end of this information sheet).

What will happen if I don't want to carry on with the study?

You can withdraw from the study at any point without giving a reason.

Will my taking part in this study be kept confidential?

All data generated by the study will be pseudonymised and any information that could identify you will replaced by an unrelated sequence of characters known as the study number. All participants will be referred to by study numbers only in any publications resulting from the study.

The Health Research Authority recommends all patient information sheets include a transparency statement relating to how the study will be conducted in accordance with the General Data Protection Regulation (GDPR) requirements. This is outlined below to explain to you who sponsors the study and how your information will be used and stored.

The University of the West of England in Bristol is the sponsor for this study. We will be using information from you in order to undertake this study and will act as the data controller for this study. This means that we are responsible for looking after your information and using it properly. The University of the West of England will keep identifiable information about you for 8 years after the study has finished. Your rights to access, change or move your information are limited, as we need to manage your information in specific ways in order for the research to be reliable and accurate. If you withdraw from the study, we will keep the information about you that we have already obtained. To safeguard your rights, we will use the minimum personally-identifiable information possible. For more information, please see the Privacy Notice that is attached to the end of this information sheet.

The University of the West of England will use your name and contact details to contact you about the research study, and make sure that relevant information about the study is recorded for your care, and to oversee the quality of the study. Individuals from The

University of the West of England and regulatory organisations may look at your medical and research records to check the accuracy of the research study. If applicable, Royal United Hospitals Bath NHS Foundation Trust, University of Southampton or Penny Brohn UK will pass these details to The University of the West of England along with the information collected from you. The only people in The University of the West of England who will have access to information that identifies you will be people who need to contact you to discuss the research or audit the data collection process. The people who analyse the information will not be able to identify you and will not be able to find out your name or contact details.

The University of the West of England will keep identifiable information about you from this study for 8 years after the study has finished.

What will happen to the results of the research study?

The study and findings will contribute to the submission of a doctoral thesis and will be disseminated via presentation at conferences and publication in academic journals. Pseudonymised direct quotations may be included in the results. The findings will be available to the public on charity websites. Participants can be given a copy of the findings if they wish.

Who is organising and funding the research?

The Chief Investigator for this study is Julie Armoogum, a Macmillan Senior Lecturer and PhD student at the University of the West of England in Bristol. The research is jointly funded by the University of the West of England and Macmillan Cancer Support.

How have patients and the public been involved with the study?

Public Research Partners, who are cancer survivors living with chronic pain, have reviewed the protocol and all participant study documentation. Any comments or suggestions from the Public Research Partners were considered, discussed with the Public Research Partners and PhD supervisory team and incorporated into the final versions where possible.

Who has reviewed the study?

- The PhD Supervisory team: Professor Candy McCabe, Professor Diana Harcourt, Professor Claire Foster and Dr Alison Llewellyn
- Public Research Partners
- An expert independent reviewer: Catherine Neck, Macmillan Cancer Rehabilitation/ Recovery Package Project Lead, Avon & Somerset
- A representative from Macmillan Cancer Support: Libby Potter, Macmillan Senior Learning and Development Manager for South West & Midlands
- A representative from Penny Brohn UK: Dr Helen Seers, Research and Evaluation Lead, Penny Brohn UK

What to expect during the consent process – what happens next?

If you would like to participate in this research, please complete the reply slip and return it to Julie Armoogum in the enclosed prepaid envelope. Alternatively, please email Julie Armoogum (Julie.armoogum@uwe.ac.uk) to register your interest.

If you are not interested, please let us know by returning the reply slip. You will not be contacted again.

If you are interested, Julie will then contact you to discuss the study in more detail, check your eligibility and answer any questions you may have. If you decide to participate in the study, a mutually convenient date will be arranged for the interview. The interview will take place face-to-face or over the telephone or Skype, dependent on your preference and practicalities of travel.

You will be asked to consent at the time of the interview. Participants having a face-to-face interview will be asked to sign a consent form. For participants having a telephone or Skype interview, verbal consent will be audio recorded.

Contact for Further Information

Chief Investigator: Julie Armoogum, Macmillan Senior Lecturer, University of the West of England.

Telephone: 0117 32 88658 Email: Julie.armoogum@uwe.ac.uk

Director of Studies: Professor Candy McCabe, Florence Nightingale Foundation Clinical Professor in Nursing, University of the West of England, Bristol & Royal United Hospitals NHS Foundation Trust, Royal National Hospital for Rheumatic Diseases, Upper Borough Walls, Bath BA1 1RL

Personal Assistant: Lindsay Davies. Telephone: 01225 473479 Email: lindsay.davies2@nhs.net

Thank you for considering participation in this study.

Privacy Notice for Research Participants

The following is the contents of the Privacy Notice that must be included with the Participant Information Sheet and Consent Form when asking participants to take part in your research: Purpose of this Privacy Notice

This statement explains how the University of the West of England (UWE, Bristol) collects, manages and uses your personal data before, during and after you participate in the research study: A qualitative exploration of the experiences and needs of cancer survivors with chronic pain. Personal data is defined as any information that can be attributed to a living individual.

We adhere to the General Data Protection Regulation (GDPR) principle of transparency. This Privacy Notice tells you about:

- How and why we use your personal data for research;

- What your rights are under GDPR, and;
- How to contact us if you have questions, concerns or wish to exercise your rights regarding the use of your personal data.

This Privacy Notice should be read in conjunction with the Participant Information Sheet and Consent Form provided to you.

Why are we processing your personal data?

UWE, Bristol undertakes research under its public function to provide research for the benefit of society. As a data controller we are committed to protecting the privacy and security of your personal data in accordance with the (EU) 2016/679 the General Data Protection Regulation (GDPR), the Data Protection Act 2018 (or any successor legislation) and any other legislation directly relating to privacy laws that apply (together "the Data Protection Legislation"). General information on Data Protection law is available from the Information Commissioner's Office (https://ico.org.uk/)

How do we use your personal data?

We use your personal data for research with appropriate safeguards in place on the lawful bases of fulfilling tasks in the public interest; and for archiving purposes in the public interest, for scientific or historical research purposes.

We will always tell you about the information we wish to collect from you and how we will use it.

Our research is governed by robust policies and procedures and where human participants are involved, is subject to ethical approval. This research has been approved by UWE's Research Ethics Committee. The research team adhere to the Ethical guidelines of the British Educational Research Association (and/or the principles of the Declaration of Helsinki, 2013) and the principles of the General Data Protection Regulation (GDPR).

For more information please see our Research Ethics webpages at: https://www1.uwe.ac.uk/research/researchethics

What data do we collect?

The data we collect will vary from project to project. Researchers will only collect data that is essential for their project.

Who do we share your data with?

We will only share your personal data in accordance with the attached Participant Information Sheet.

How do we keep your data secure?

We take a robust approach to protecting your information with secure electronic and physical storage areas for research data with controlled access. If you are participating in a particularly sensitive project the University puts into place additional layers of security. The University has Cyber Essentials information security certification.

Alongside these technical measures there are comprehensive and effective policies and processes in place to ensure that users and administrators of information are aware of their obligations and responsibilities for the data they have access to. By default, people are only granted access to the information they require to perform their duties. Mandatory data protection and information security training is provided to staff and expert advice available if needed.

How long do we keep your data for?

Your personal data will only be retained for as long as is necessary to fulfil the cited purpose of the research. The length of time we keep your personal data will depend on several factors including the significance of the data, funder requirements, and the nature of the study. Specific details are provided in the attached Participant Information. Anonymised data may be stored in UWE's research data archive or another carefully selected appropriate data archive.

Your rights and how to exercise them

Under the Data Protection legislation you have the following qualified rights:

(1) The right to access your personal data held by or on behalf of the University;

(2) The right to rectification if the information is inaccurate or incomplete;

(3) The right to restrict processing and/or erasure of your personal data;

(4) The right to data portability;

(5) The right to object to processing;

(6) The right to object to automated decision making and profiling;

(7) The right to complain to the Information Commissioner's Office (ICO).

Please note that some of these rights do not apply when the data is being used for research purposes if appropriate safeguards have been put in place.

We will always respond to concerns or queries you may have. If you wish to exercise your rights or have any other general data protection queries, please contact the Data Protection Officer. (dataprotection@uwe.ac.uk).

If you have any complaints or queries relating to the specific research you are taking part in please contact either the research lead, whose details are in the attached Participant Information Sheet, UWE's Research Ethics Committee (research.ethics@uwe.ac.uk) or UWE's research governance manager (Ros.Rouse@uwe.ac.uk)

Professor Lauren Devine, November 2018

Appendix D- Reply slip

UWE headed paper

Reply slip **Study Number…………………………………..**

☐ Yes, I would like to discuss taking part in the study or receive more information.

Name………
……………

Please provide your telephone number so I can call you:

Telephone number………

☐ No, I would not like more information about this study.

We would be grateful if you could explain why:

I do not feel I am suitable for this study ☐ I do not want to be interviewed ☐

Other………
………

Please return this form in the prepaid envelope provided to:

Julie Armoogum, Macmillan Senior Lecturer, UWE Bristol, 2B28, Glenside Campus, Blackberry Hill, Bristol, BS16 1DD or email Julie.armoogum@uwe.ac.uk

Thank you for considering participation in this study.

Protocol: The experiences and needs of cancer survivors with chronic pain. Version 2. 07.08.19. IRAS number 255086

Appendix E- Consent form

IRAS ID: 255086

CONSENT FORM

Participant Information Sheet: An exploration of the experiences and needs of cancer survivors with chronic pain.

1. I confirm that I have read the information sheet dated XXX (version X) for the above study. I have had the opportunity to consider the information, ask questions and have had these answered satisfactorily. ☐

2. I understand that my participation is voluntary and that I am free to withdraw at any time without giving any reason, without my medical care or legal rights being affected. ☐

3. I understand that the information collected about me will be used to support other research in the future, and may be shared anonymously with other researchers. ☐

4. I understand I may be contacted in the future to consider participating in further research that forms part of this doctoral study. ☐

5. I agree to my General Practitioner being informed of my participation in the study. ☐

6. I would like to be informed of the findings of the study ☐

7. I agree to take part in the above study ☐

____________________	____________________	____________________
Name of Participant	Date	
____________________	____________________	____________________
Name of Person taking consent	Date	Signature

One copy of this form will be sent to participants, one copy will be scanned on a UWE owned and managed scanner and stored in a password protected folder on a UWE password protected OneDrive cloud storage facility. Paper copies will then be securely destroyed.

Appendix F - Follow up letters: 1) For centre 1 2) For centre 2 3) For centre 3 and 4) for participants who responded to the social media campaign.

1. RNHRD headed paper

Dear……

A few weeks ago we wrote to you to let you know about a research study exploring chronic pain in cancer survivors.

A team of researchers at the University of the West of England in Bristol are exploring the experiences and needs of cancer survivors with chronic pain. In this study, researchers are particularly interested in the experiences of people who have completed their cancer treatment as an adult and their cancer has gone, but who are living with chronic pain as a consequence of their cancer treatment.

You may feel that you would like find out more about this research. If so, please complete the enclosed reply slip and return it in the prepaid envelope to Julie Armoogum, Macmillan Senior Lecturer, University of the West of England. Alternatively, you can email Julie.armoogum@uwe.ac.uk or telephone 0117 32 88658.

If you are not interested, we would be very grateful if you would complete the reply slip to let us know and then we will know not to contact you again.

With very best wishes,

Professor Candy McCabe

Protocol: The experiences and needs of cancer survivors with chronic pain. Version 2. 07.08.19. IRAS number 255086

Professor Candy McCabe, Florence Nightingale Foundation Clinical Professor in Nursing, University of the West of England, Bristol & Royal United Hospitals NHS Foundation Trust, Royal National Hospital for Rheumatic Diseases, Upper Borough Walls, Bath BA1 1RL

Personal Assistant: Lindsay Davies. Telephone: 01225 473479 Email: lindsay.davies2@nhs.net

2. Penny Brohn headed paper

Dear……

A few weeks ago we wrote to you to let you know about a research study exploring chronic pain in cancer survivors.

A team of researchers at the University of the West of England in Bristol are exploring the experiences and needs of cancer survivors with chronic pain. In this study, researchers are particularly interested in the experiences of people who have completed their cancer treatment as an adult and their cancer has gone, but who are living with chronic pain as a consequence of their cancer treatment.

You may feel that you would like find out more about this research. If so, please complete the enclosed reply slip and return it in the prepaid envelope to Julie Armoogum, Macmillan Senior Lecturer, University of the West of England. Alternatively, you can email Julie.armoogum@uwe.ac.uk or telephone 0117 33 88658.

If you are not interested, we would be very grateful if you would complete the reply slip to let us know and then we will know not to contact you again.

With very best wishes,

Dr Marian Naidoo

Director of Services, Penny Brohn UK

3. Southampton headed paper

Dear……

A few weeks ago we wrote to you to let you know about a research study exploring chronic pain in cancer survivors.

A team of researchers at the University of the West of England in Bristol are exploring the experiences and needs of cancer survivors with chronic pain. In this study, researchers are particularly interested in the experiences of people who have completed their cancer treatment as an adult and their cancer has gone, but who are living with chronic pain as a consequence of their cancer treatment.

You may feel that you would like find out more about this research. If so, please complete the enclosed reply slip and return it in the prepaid envelope to Julie Armoogum, Macmillan Senior Lecturer, University of the West of England. Alternatively, you can email Julie.armoogum@uwe.ac.uk or telephone 0117 33 88658.

If you are not interested, we would be very grateful if you would complete the reply slip to let us know and then we will know not to contact you again.

With very best wishes,

Professor Claire Foster

Professor of Psychosocial Oncology, Director of Macmillan Survivorship Research Group

University of Southampton

4. UWE headed paper

Dear……

A few weeks ago we wrote to you to let you know about a research study exploring chronic pain in cancer survivors.

A team of researchers at the University of the West of England in Bristol are exploring the experiences and needs of cancer survivors with chronic pain. In this study, researchers are particularly interested in the experiences of people who have completed their cancer treatment as an adult and their cancer has gone, but who are living with chronic pain as a consequence of their cancer treatment.

You may feel that you would like find out more about this research. If so, please complete the enclosed reply slip and return it in the prepaid envelope to Julie Armoogum, Macmillan Senior Lecturer, University of the West of England. Alternatively, you can email Julie.armoogum@uwe.ac.uk or telephone 0117 33 88658.

If you are not interested, we would be very grateful if you would complete the reply slip to let us know and then we will know not to contact you again.

With very best wishes,

Julie Armoogum

Macmillan Senior Lecturer

University of the West of England

Appendix G: Eligibility screening tool

**Study number**		
Person confirming eligibility		
	**Yes**	**No**
Over 18 at diagnosis and treatment		
Completed anti-cancer therapy		
Pain lasting for 3 months of more		
No known active disease		
**Eligible for study**		

Appendix H: GP letter

4. UWE Bristol headed paper

Dear……

We would like to inform you that your patient……………. has agreed to participate in a research study called: A qualitative exploration of the experiences and needs of cancer survivors with chronic pain.

The research study fits into a programme of work that aims to establish healthcare needs of cancer survivors with chronic pain and review how well current services meet their needs. The work will be submitted as a doctoral thesis and the PhD supervisory team includes Professor Candy McCabe (University of the West of England, Bristol), Professor Diana Harcourt (University of the West of England, Bristol), Professor Claire Foster (University of Southampton) and Dr Alison Llewellyn (University of the West of England, Bristol).

If you would like more information about the research, please do not hesitate to contact me:

Julie Armoogum, Macmillan Senior Lecturer, University of the West of England, 2B28, Glenside Campus, Blackberry Hill, Bristol, BS16 1DD

Telephone: 0117 32 88658 Email: Julie.armoogum@uwe.ac.uk

With very best wishes,

Julie Armoogum

Macmillan Senior Lecturer

University of the West of England

Appendix I: Interview schedule

- Welcome, introductions, brief questions to build rapport and put participant at ease (travel, comfort, check seating and temperature etc).
- Explain purpose of interview - part of a programme of looking at experiences, needs and service provision for cancer survivors with chronic pain. We'll start with some brief demographic questions and then we'll talk about your experiences of cancer and your chronic pain. After that, we'll move on to the support you have received to help with your pain and any support you think would be or would have been helpful.
- Confirm consent.
- **Experiences**
- Can you tell me about your cancer diagnosis and treatment? (if needed, probe for type of cancer, time since treatment has finished and if had chemotherapy, radiotherapy, surgery, hormone therapy or other types of treatment. Ask about age at diagnosis and age now)
- And how have things been since?
- When did your pain related to the cancer treatment first start?
- Can you tell me more about what it's been like living with the pain?
- What were you told about the risks of chronic pain?
- Probing questions as participant talks: Can you tell me more? Can you describe? What do you think? What do you feel? Can you reflect? What else is of importance?
- In general have you felt supported in coping with your treatment related pain?
- **Services**
- Moving onto look at service provision…
- What services have supported you with your pain?
- How did you access those services?
- Did you have problems in accessing the services?
- What do you think worked well? Less well? What hindered or helped?
- Can you tell me a bit more about that? How did you feel about that?
- What would have been helpful to you at the beginning?
- What would be helpful for you now?

Draw interview to close. Thank for time. Offer support leaflet.

Appendix J: Support leaflet for participants

Thank you for participating in this research. We really value your views and are very grateful for your time.

If you would like to talk through any issues that may have arisen by taking part in this research, please contact your G.P, care team or one of the charities listed below.

Macmillan Cancer Support – Telephone: 0808 808 00 00. Lines are open seven days a week, 8am –to 8pm and it is free to call.

The Website offers lots of information and practical advice and support:

https://www.macmillan.org.uk/information-and-support

Maggie's Centres – Telephone: 0300 123 1801. There are Maggie's Centres nationwide that offer free practical, emotional and social support to people with cancer and their families and friends.

The website gives details of the centres and also offers an Online Centre for practical, emotional and social support:

https://www.maggiescentres.org/how-maggies-can-help/help-available/

Penny Brohn UK – Telephone: 0303 3000 118. Penny Brohn UK has a national centre in Bristol and works in partnership with other healthcare organisations to provide support across the UK.

Penny Brohn supports a Whole Life Approach and focuses on building resilience in every aspect of life and supporting the body's natural ability to heal and repair itself. The website has lots of information and support and details of course they run:

https://www.pennybrohn.org.uk/
