# Supplementary material for: ‘I think it affects every aspect of my life, really’: Cancer survivors’ experience of living with chronic pain after curative cancer treatment in England, UK
Source: PLoS One. 2023 Sep 1;18(9):e0290967. doi: 10.1371/journal.pone.0290967 (PMC10473538; doi:10.1371/journal.pone.0290967)
Supplement: S2 File — (PDF) [file pone.0290967.s002.pdf]

Interview schedule

- **Audio part 1**
-
- Welcome, introductions, brief questions to build rapport and put participant at ease (travel, comfort, check seating and temperature etc).
- Explain purpose of interview - part of a programme of looking at experiences, needs and service provision for cancer survivors with chronic pain. We'll start with some brief demographic questions and then we'll talk about your experiences of cancer and your chronic pain. After that, we'll move on to the support you have received to help with your pain and any support you think would be or would have been helpful.
- Confirm consent and check GP

- **Audio part 2**

- **Experiences**
- Can you tell me about your cancer diagnosis and treatment? (if needed, probe for type of cancer, time since treatment has finished and if had chemotherapy, radiotherapy, surgery, hormone therapy or other types of treatment. Ask about age at diagnosis and age now)
- And how have things been since?
- When did your pain related to the cancer treatment first start?
- Can you tell me more about what it's been like living with the pain?
- What were you told about the risks of chronic pain?
- Probing questions as participant talks: Can you tell me more? Can you describe? What do you think? What do you feel? Can you reflect? What else is of importance?
- In general have you felt supported in coping with your treatment related pain?
- **Services**
- Moving onto look at service provision…
- What services have supported you with your pain?
- How did you access those services?
- Did you have problems in accessing the services?

- What do you think worked well? Less well? What hindered or helped?
- Can you tell me a bit more about that? How did you feel about that?
- What would have been helpful to you at the beginning?
- What would be helpful for you now?

Draw interview to close. Thank for time. Offer support leaflet.
